# Structural-functional network decoupling in early stage amyotrophic lateral sclerosis reveals cell-type specific transcriptional signatures

**DOI:** 10.1186/s12916-026-04932-7

**Published:** 2026-07-02

**Authors:** Jixin Luan, Yan Yun, Yichang Jiao, Yao Wang, Mingjie Ma, Didi Shan, Hongxu Wang, Xinbo Ji, Yao Tang, Jianing Li, Zexin Zhan, Xiaohan Sun, Ninglu Gao, Pengfei Lin, Chuanzhu Yan, Dexin Yu, Shuangwu Liu, Fuchen Liu

**Affiliations:** 1 Department of Neurology, Research Institute of Neuromuscular and Neurodegenerative Diseases, Shandong Key Laboratory of Mitochondrial Medicine and Rare Diseases, Qilu Hospital of Shandong University, Jinan, 250012 Shandong China; 2 Department of Radiology, Qilu Hospital of Shandong University, Jinan, 250012 Shandong China; 3 Department of Radiology, Shandong Cancer Hospital and Institute, Shandong First Medical University, Shandong Academy of Medical Sciences, Jinan, 250117 Shandong China; 4 School of Nursing and Rehabilitation, Cheeloo College of Medicine, Shandong University, Jinan, 250012 Shandong China

**Keywords:** Amyotrophic lateral sclerosis, Structural-functional coupling, Transcriptional patterns, Single cell, Microglia

## Abstract

**Background:**

Amyotrophic lateral sclerosis (ALS) involves widespread brain network dysfunction, yet the molecular mechanisms linked to these alterations remain poorly understood. We investigated macroscopic structural-functional coupling abnormalities in early-stage ALS (ALS-ES) and their underlying transcriptomic signatures.

**Methods:**

We analyzed multimodal MRI data from 73 patients with sporadic ALS-ES and 74 age- and sex-matched healthy controls. Structural-functional (SC-FC) coupling was quantified using diffusion tensor imaging and resting-state functional MRI. Machine learning models were constructed to distinguish patients from controls based on network features. Coupling alterations were spatially correlated with neurotransmitter receptor maps and gene expression profiles from the Allen Human Brain Atlas. Key transcriptomic findings were validated using independent single-cell RNA sequencing datasets.

**Results:**

While structural connectivity remained largely preserved, functional connectivity was significantly reduced in the somatomotor network (SMN). This mismatch manifested as significant SC-FC network decoupling, particularly within the SMN (*p*_FDR_ = 0.001). A gradient boosting machine model accurately classified patients, identifying SC-FC coupling in the left precentral gyrus as a primary statistical contributor to the classification model. Decoupling spatially correlated with 5-HT2A and mGluR5 receptor distributions. Imaging-transcriptomics linked network failure to a gene signature enriched for synaptic pathways and microglial markers. Single-cell analysis identified FMN1 as a candidate gene whose glial expression spatially associates with network decoupling.

**Conclusions:**

Early-stage ALS is characterized by significant structural-functional network decoupling, primarily in motor systems. This macroscopic failure is linked to specific microglial dysregulation, particularly FMN1 downregulation, providing a multiscale framework bridges statistical neuroimaging signatures with potential cellular pathology.

**Supplementary Information:**

The online version contains supplementary material available at https://doi.org/10.1186/s12916-026-04932-7.

## Background

Amyotrophic lateral sclerosis (ALS) is a rapidly progressive and fatal neurodegenerative disease characterized by the progressive loss of motor neurons [1]. While the Gold Coast criteria provide the current standard for diagnostic certainty [2], clinical staging via the King’s system offers a validated framework for identifying the earliest symptomatic phases. Patients at King’s Stage 1, characterized by clinical involvement limited to a single region, represent a critical population for studying early-stage ALS (ALS-ES) [3]. While historically considered a motor system disorder, evidence now confirms that early stage ALS involves widespread brain network dysfunction, including alterations in cognitive and non-motor symptoms [4, 5]. Therefore, it is crucial to explore abnormalities in macroscopic brain networks in ALS-ES and their underlying molecular biological basis.

Traditional neuroimaging studies have identified diverse cerebral structural and functional alterations in patients with ALS [6, 7]. However, most research has focused on single-modal features, overlooking an integrated perspective on how structural integrity supports functional activity. Structure-function coupling quantifies the relationship between white matter fiber connectivity patterns (structural connectivity, SC) and their corresponding functional communication strength (functional connectivity, FC) [8]. Altered SC-FC coupling in ALS may reflect a crucial imbalance between structural damage and functional reorganization [9]. However, the multimodal, high-dimensional data generated from SC, FC, and SC-FC coupling present a significant analytical challenge. Machine learning algorithms provide powerful tools to address this, capable of integrating these complex imaging features to build robust classification models for distinguishing patients from healthy controls and identifying key predictive biomarkers.

While machine learning can identify which features are predictive, the underlying molecular, cellular, and transcriptomic mechanisms associated with these SC-FC coupling changes remain unclear. Imaging-transcriptomics has emerged as a significant frontier, using the Allen Human Brain Atlas (AHBA) to link macroscopic network abnormalities with molecular mechanisms [10]. This methodology has successfully been applied to neurodegenerative conditions like Alzheimer’s disease [11] and Parkinson’s disease [12], but this integrative approach remains underexplored in early stage ALS. Given that ALS involves the motor system and extensive cortical pathways, altered SC-FC coupling likely reflects a complex interplay between the loss of structural integrity due to neuronal degeneration and the subsequent functional network reorganization [13]. Identifying the gene expression profiles associated with altered SC-FC can pinpoint critical molecular signatures underlying network collapse. Critically, ALS pathology is not purely neuronal but also involves substantial dysfunction of glial cells [14]. Bulk-tissue transcriptomic analyses are insufficient to pinpoint which specific cell types are responsible for the observed gene expression changes. Therefore, single-cell analysis techniques are urgently needed to resolve this cellular heterogeneity and elucidate how dysfunction in specific cell populations relates to macroscopic network decoupling.

Building on this background, this study aims to comprehensively investigate SC-FC coupling alterations in early-stage ALS patients by integrating multimodal data. Our approach will first quantify differential SC-FC coupling patterns across global, regional, and network levels. We will then apply machine learning models to these multimodal features to build a diagnostic model. To uncover the underlying mechanisms, we will evaluate correlations between coupling abnormalities and spatial maps of neurotransmitter receptors and cognitive functions. Furthermore, we will use imaging-transcriptomics (PLS regression) to identify associated gene expression profiles, and subsequently use bioinformatic enrichment and single-cell data validation to elucidate the specific biological pathways, cortical layers, and cell types involved. This multi-scale integration, from macroscopic networks to single-cell resolution, is expected to provide novel evidence for the complex multiscale associations of early-stage ALS.

## Methods

### Study Participants and Data Acquisition

**Fig. 1:**
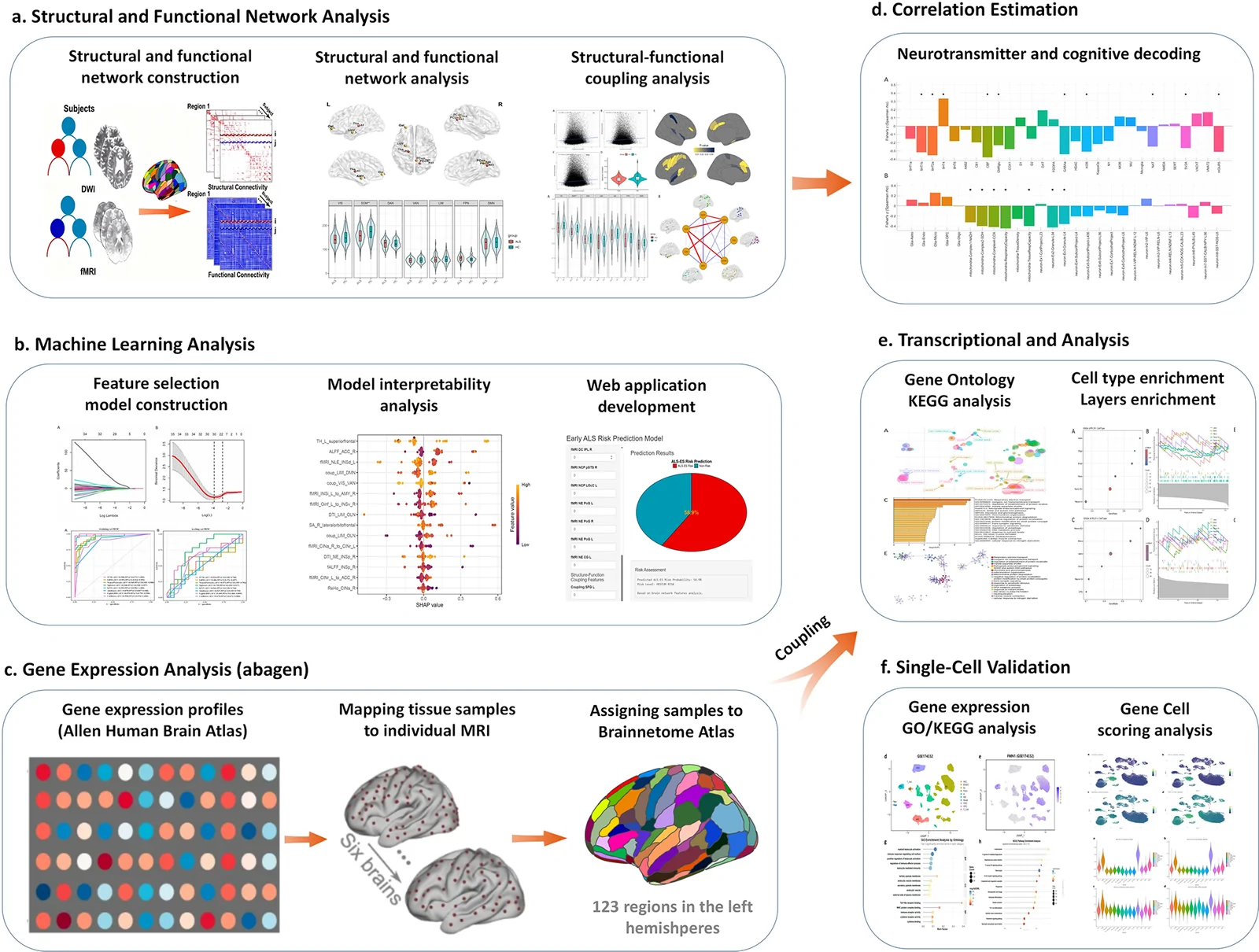
The flowchart of this study. **a** Structural-Function Coupling Analysis: DTI and fMRI data were used to construct structural (SC) and functional (FC) networks. SC-FC coupling was then quantified at global, regional, and network levels. **b** Machine Learning Analysis: Multimodal network features were selected using LASSO regression. Eight models were trained to classify ALS, with the best model interpreted using SHAP. A web-based prediction app was also developed. **c** Gene Expression Analysis: Gene expression data from the Allen Human Brain Atlas (AHBA) was mapped to the Brainnetome Atlas using the abagen toolbox. PLS regression identified genes associated with SC-FC coupling. **d** Correlation Estimation: The SC-FC alteration map was spatially correlated with neurotransmitter distributions (JuSpace). **e** Transcriptional Analysis: PLS-identified gene sets were analyzed for GO/KEGG pathways, EWCE and ssGSEA. **f** Single-Cell Validation: Public single-cell datasets (GSE219281, GSE174332) were analyzed using Seurat to validate key genes (e.g., FMN1) and pathways in specific cell types

Seventy-three patients with sporadic early stage ALS (ALS-ES) meeting Gold Coast criteria and 74 age- and sex-matched healthy controls (HCs) were enrolled in this case-control study [15]. To ensure a rigorous definition of the cohort, all patients were first diagnosed with ALS according to the Gold Coast criteria to establish diagnostic certainty. Subsequently, patients were strictly selected based on the King’s staging system, with only those at King’s Stage 1 included in the ALS-ES group. King’s Stage 1 is defined by the involvement of only a single clinical domain (bulbar, upper limb, or lower limb), which serves as a validated marker for the early symptomatic phase of the disease. All participants underwent comprehensive clinical assessment including the revised ALS Functional Rating Scale (ALSFRS-R) [16], Hamilton Depression Rating Scale (HDRS), and Hamilton Anxiety Rating Scale (HARS) [17]. Exclusion criteria encompassed familial ALS, significant neurological comorbidities, and inability to complete magnetic resonance imaging (MRI) protocols. This study was approved by the Research Ethics Committee of the School of Medicine, Shandong University. Participant information was collected only after all patients and HCs had been made aware of the purpose of the study and provided their written informed consent. Detailed demographic and clinical characteristics are summarized in Table 1. Multimodal MRI data were acquired using a 3.0T Philips Ingenia scanner, including high-resolution T1-weighted, resting-state functional MRI (rs-fMRI), and diffusion-tensor imaging (DTI) sequences. Total intracranial volume (TIV) was estimated via the FreeSurfer 6.0 recon-all pipeline as a covariate for all subsequent statistical models. Complete MRI acquisition parameters are provided in Additional file 1: Methods S1. The flowchart is shown in Fig. 1.

**Table 1:** Clinical data of ALS-ES and healthy controls

Variables	ALS-ES (*n* = 73)	HCs (*n* = 74)	*p*
Age (years)	55.64 ± 11.87	54.93 ± 6.89	0.658
Sex (Male/Female)	42 / 31	31 / 43	0.083
Bulbar onset, n (%)	17 (23.3%)	-	-
ALS duration (months)	11.7 ± 8.0	-	-
ALSFRS-R	40.8 ± 3.4	-	-
HARS (Median[IQR])	10 [4–18]	5 [1–7]	*p*<0.01
HDRS (Median[IQR])	8 [3–13]	2 [2–4]	*p*<0.01
TIV (dm^3^)	1.401 ± 0.180	1.380 ± 0.217	0.517
meanFD	0.233 ± 0.122	0.225 ± 0.094	0.640

*ALS-ES* early stage amyotrophic lateral sclerosis, *HCs* healthy controls, *ALSFRS-R* Amyotrophic Lateral Sclerosis Functional Rating Scale-Revised, *HARS* Hamilton Anxiety Rating Scale, *HDRS* Hamilton Depression Rating Scale, *TIV* Total Intracranial Volume, *meanFD* Mean Framewise Displacement

### Structural Brain Network Construction and Analysis

Structural connectivity matrices were constructed from DTI data processed using the Pipeline for Analyzing Brain Diffusion Images (PANDA) toolbox. Preprocessing included eddy current correction, motion artifact removal, and tensor estimation. Whole-brain deterministic tractography was performed using the Fiber Assignment by Continuous Tracking (FACT) algorithm with termination criteria of fractional anisotropy (FA) < 0.2 or angle > 45°. Following established protocols in recent connectomics research [18, 19], this deterministic approach was selected to provide a stable and conservative macroscale anatomical framework for SC-FC coupling analysis. Network nodes were defined using the Brainnetome Atlas (BNA-246), with edges weighted by the product of FA and fiber number (FN) (threshold: FN ≥ 3, prevalence ≥ 75%). The FN × FA product served as a robust composite measure of macroscale connectivity strength, ensuring a reliable structural backbone that minimizes potential false-positive connections. The 210 cortical regions were subsequently assigned to seven functional networks based on the Yeo template [20], including the visual network (VIS), somatomotor network (SMN), dorsal attention network (DAN), ventral attention network (VAN), limbic network (LIM), frontoparietal network (FPN), and default mode network (DMN). Comprehensive preprocessing pipelines are detailed in Additional file 1: Methods S2.

### Functional Brain Network Construction and Analysis

Functional connectivity matrices were derived from preprocessed rs-fMRI data using Graph Theoretical Network Analysis (GRETNA) toolbox, involving motion correction, band-pass filtering (0.01–0.10 Hz), and regression of nuisance covariates. To ensure spatial consistency across modalities, network nodes were defined using the same BNA-246 atlas as in the structural analysis. Pearson correlation coefficients between regional time series were computed across sparsity thresholds (0.05–0.50), with area under the curve values serving as integrated connectivity measures. Consistent with the structural framework, the 210 cortical regions were assigned to seven functional networks according to the Yeo template, allowing for the assessment of intra-network and inter-network functional organization. Comprehensive preprocessing pipelines are detailed in Additional file 1: Methods S3.

### SC-FC Coupling Analysis

Structure-function coupling was quantified using Spearman correlation to account for the significant non-Gaussian and heavy-tailed distribution of structural connectivity weights. This rank-based approach is a robust methodology validated in prior connectomics research [18, 21, 22], as it minimizes the influence of high-weight outliers and effectively captures monotonic relationships in brain networks. Global coupling represented the correlation between all non-zero SC weights and corresponding FC values. Regional coupling was computed as node-wise correlations between SC and FC profiles. Network-level analysis assessed both intra-network (within networks) and inter-network (between networks) coupling based on seven canonical functional networks defined by the Yeo template. Group differences were examined using general linear models controlling for age, sex and intracranial total volume (TIV). To ensure the robustness of our findings against potential confounders, we performed sensitivity analyses for clinical heterogeneity using ANCOVA for subgroup comparisons and partial correlation for clinical associations. Crucially, these sensitivity models were adjusted for a comprehensive set of covariates, including age, sex, TIV, mean framewise displacement (FD), HARS, and HDRS scores, to confirm that the observed SC-FC coupling alterations were independent of affective symptoms and head motion. Complete analytical specifications are provided in Additional file 1: Methods S4.

### Machine Learning Analysis

To classify ALS-ES patients from HCs, we implemented a machine learning pipeline using features from structural, functional, and SC-FC analyses. To ensure the integrity of performance estimates, all group-dependent preprocessing steps, including feature selection and data normalization, were strictly nested within each cross-validation fold. A two-stage feature selection procedure, comprising a t-test filter (*p* < 0.01) followed by least absolute shrinkage and selection operator (LASSO) regression, was conducted independently on the training set of each fold to prevent circular reasoning and data leakage. The resulting features are characterized as predictive signatures that optimize classification performance within this cohort. We trained eight algorithms, such as Support Vector Machine (SVM) and eXtreme Gradient Boosting (XgBoost), with hyperparameters optimized through ten-fold cross-validation and random search. Model performance was assessed using AUC, accuracy, and other metrics, with 1000 bootstrap repetitions for AUC robustness and Decision Curve Analysis (DCA) for clinical utility [23]. The optimal model was interpreted using SHapley Additive exPlanations (SHAP) to enhance transparency. Additionally, a web-based prediction application was developed using Shiny to demonstrate the model’s practical application. To assess the incremental value of imaging markers, a clinical baseline model was constructed using age and sex as predictors. Both the imaging and clinical models were evaluated using an identical machine learning framework. Comprehensive methodological details are available in Additional file 1: Methods S5-S7.

### Neurotransmitter and Cognitive Correlates of SC-FC Coupling

Spatial correlations between SC-FC coupling alterations and neurotransmitter systems were evaluated using the JuSpace toolbox [24]. Spearman correlations across neuromorphometrics atlas regions were computed for 56 neurotransmitter receptors/transporters, with significance assessed via permutation testing (10,000 iterations) and false discovery rate (FDR) correction. Cognitive decoding was performed using the Neurosynth platform to identify meta-analytically derived cognitive terms associated with SC-FC patterns [25–27]. Detailed methodological descriptions are available in Additional file 1: Methods S8.

### Imaging-Transcriptomics Analysis

Regional gene expression data from the Allen Human Brain Atlas were processed using the abagen toolbox and mapped to left-hemisphere BNA regions [28]. This framework implements a standardized pipeline to account for regional sampling density and donor-specific variations through weighted averaging and distance-dependent consistency checks [28, 29]. Partial least squares (PLS) regression identified genes whose expression covaried with SC-FC coupling patterns. These associations were interpreted as markers of baseline regional vulnerability. The first PLS component (PLS1) explaining maximal covariance was retained for subsequent analysis. Genes with significant weights (*p*_Bonferroni_ <0.05) were categorized as PLS1+ (positive association) or PLS1- (negative association). To account for spatial autocorrelation, all imaging-transcriptomic correlations were assessed using spatial permutation tests (spin tests, 10,000 iterations), ensuring results are robust against random spatial patterns. Complete processing pipelines are described in Additional file 1: Methods S9.

### Functional and Cellular Annotation

To characterize the biological significance of the identified PLS1 + and PLS1- gene sets, we performed functional enrichment and cellular specificity analyses. Gene Ontology (GO) and Kyoto Encyclopedia of Genes and Genomes (KEGG) pathway annotations were conducted using the clusterProfiler R package [30] and the Metascape online platform. Furthermore, we investigated cell-type specificity using expression weighted cell type enrichment (EWCE) analysis [31] with two independent human single-cell RNA sequencing datasets(AHBA-seq [32] and DroNc-seq [33]). Cortical layer enrichment was quantified across layers I-VI using single-sample gene set enrichment analysis (ssGSEA) [34]. Comprehensive bioinformatic methodologies are detailed in Additional file 1: Methods S10.

### Single-Cell Analysis

Single-cell RNA sequencing data from motor cortex (GSE219281 [35], GSE174332 [36]) were analyzed using Seurat. Quality control excluded cells with < 200 or > 10,000 genes, or > 20% mitochondrial/ribosomal gene content. Following normalization and scaling, we performed principal component analysis (PCA), t-distributed stochastic neighbor embedding (t-SNE), uniform manifold approximation and projection (UMAP), and clustering. Cell types were annotated using SingleR. We examined expression patterns of PLS-derived gene sets correlated with SC-FC coupling across cell types in ALS versus controls, followed by GO, KEGG enrichment analyses, and module scoring. Comprehensive analytical procedures are detailed in Additional file 1: Methods S11.

## Results

### Demographic Information and Clinical Characteristics

Table 1 summarizes and compares the demographic and clinical characteristics between patients with early-stage amyotrophic lateral sclerosis (ALS-ES, *n* = 73) and healthy controls (HCs, *n* = 74). The two groups were well-matched in terms of age (*p* = 0.658) and sex distribution (*p* = 0.083), supporting the comparability of the cohorts. Among the ALS-ES patients, 23.3% (*n* = 17) presented with bulbar-onset disease. The ALS-ES group exhibited a mean ALSFRS-R score of 40.8 ± 3.4 and a mean disease duration of 11.7 ± 8.0 months. Crucially, the entire patient cohort was at King’s Stage 1, representing the earliest clinical phase of the disease characterized by isolated regional involvement. Significantly higher scores were observed in ALS-ES patients compared to HCs on both the HARS (*p* < 0.01) and the HDRS (*p* < 0.01), reflecting greater levels of anxiety and depressive symptoms in the patient group.

### Network Alterations in Early-stage ALS

**Fig. 2:**
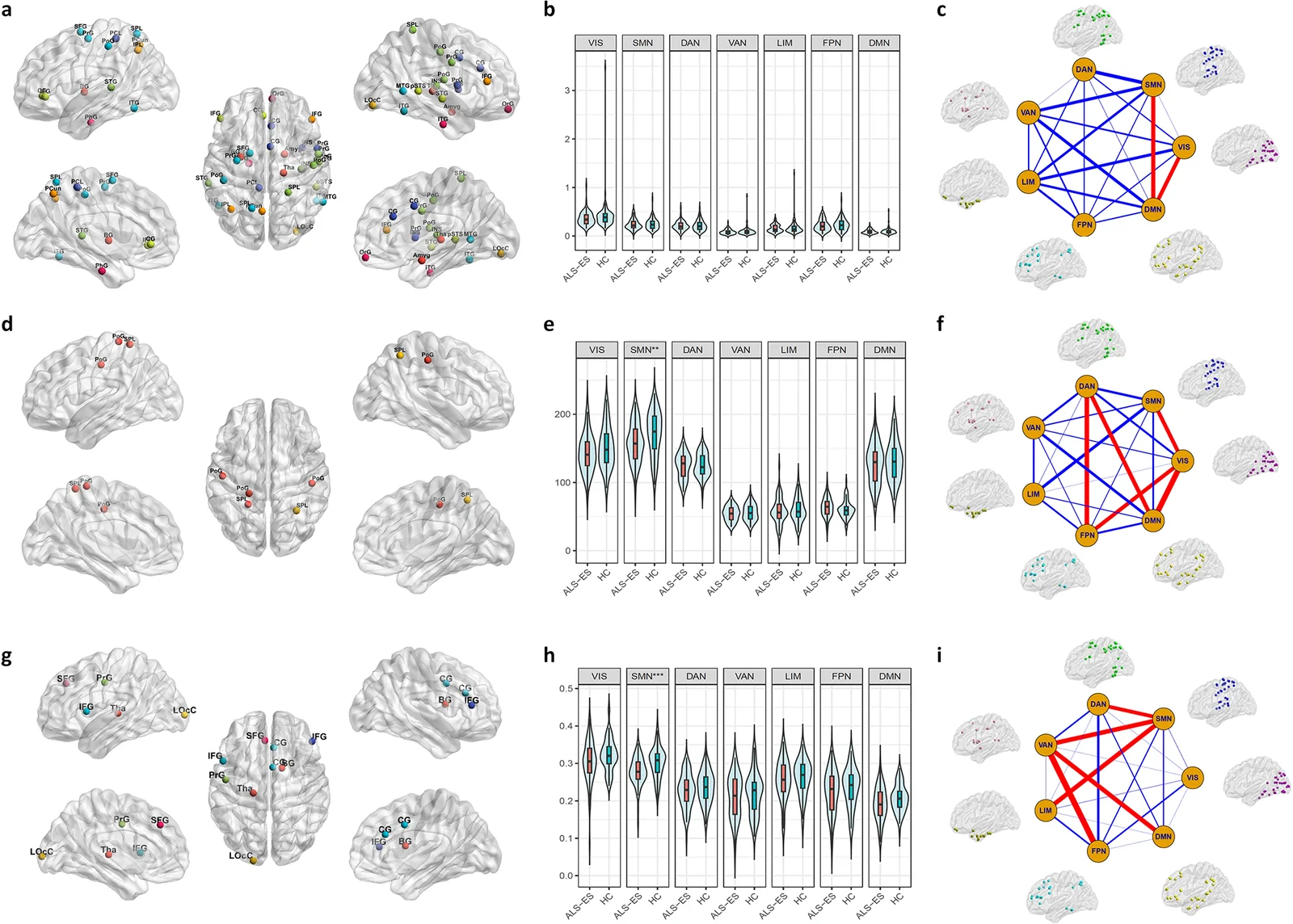
Analysis of structural connectivity, functional connectivity, and structural-functional coupling. **a** Brain regions showing nominal (uncorrected *p* < 0.05) alterations in structural nodal topological properties. **b** Violin plots of intra-network structural connectivity. **c** Circle plot of t-values for inter-network structural connectivity. Line thickness represents the absolute t-value, and red lines indicate network pairs with nominally significant group differences (uncorrected *p* < 0.05). **d** Brain regions showing significant (*p*_*FDR*_ < 0.05) alterations in functional nodal topological properties. **e** Violin plots of intra-network functional connectivity, showing a significant reduction in the SMN. **f** Circle plot of t-values for inter-network functional connectivity. Red lines indicate network pairs with nominally significant group differences (uncorrected *p* < 0.05). **g** Brain regions showing significant (*p*_*FDR*_ < 0.05) alterations in SC-FC coupling. **h** Violin plots of intra-network SC-FC coupling, showing a significant reduction in the SMN. **i** Circle plot of t-values for inter-network SC-FC coupling. Red lines indicate network pairs with nominally significant group differences (uncorrected *p* < 0.05)

Structural network analysis revealed no significant group differences in global (Additional file 1: Table S1) or nodal (Additional file 1: Table S2) topological properties after FDR correction. While nominal nodal differences were widespread, no regions survived multiple comparisons. Similarly, both intra-network (Additional file 1: Table S3) and inter-network (Additional file 1: Table S4) structural connectivity, quantified as macroscale connectivity strength via the product of FA and FN, remained statistically stable in the ALS-ES group, though uncorrected decreases were noted between the visual network and default mode network (VIS-DMN), the somatomotor network and default mode network (SMN-DMN) (Fig. 2a-c). In contrast, functional network analysis identified significant alterations despite unchanged global metrics (Additional file 1: Table S5). At the nodal level (Fig. 2d), the ALS-ES group showed significant disruptions (*p*_*FDR*_ < 0.05) in the parietal lobe, including altered betweenness centrality in the right superior parietal lobule (SPL) and postcentral gyrus (PoG), and decreased nodal efficiency and degree centrality in the left SPL and PoG (Additional file 1: Table S6). At the network level, intra-network functional connectivity was significantly reduced in the SMN (*p*_*FDR*_ = 0.008) (Fig. 2e; Additional file 1: Table S7). Finally, several inter-network functional connections, including those between the visual network and default mode network (VIS-DMN) and between the dorsal attention network and default mode network (DAN-DMN), exhibited nominal decreases that did not survive FDR correction (Fig. 2f; Additional file 1: Table S8) (See Additional file 1: Result S1 and S2 for details).

### Structural-Functional Coupling Alterations in ALS-ES

Analysis of SC-FC coupling revealed no significant group difference at the global level (*p* = 0.37). However, ALS-ES patients exhibited significant regional SC-FC coupling reductions (*p*_*FDR*_ < 0.05) in several areas, including the left superior frontal gyrus (SFG_L_7_6), left precentral gyrus (PrG_L_6_1), and right cingulate gyrus (CG_R_7_5) (Fig. 2g, Additional file 1: Table S9). At the network level, intra-network SC-FC coupling was significantly decreased in the SMN (*p*_*FDR*_ = 0.001) (Fig. 2h, Additional file 1: Table S10). Effect size analysis revealed that the magnitude of SC-FC decoupling was highest in the SMN (Cohen’s *d* = 0.605), nearly doubling the average effect size seen across other functional networks (mean *d* = 0.230), further supporting the regional vulnerability of the motor system (Additional file 1: Fig. S1a**)**. The robustness of the SMN decoupling was further validated using a permutation test (5,000 iterations), confirming that the observed reduction was significantly greater than chance (T = 2.833, *p*_perm_ = 0.004) (Additional file 1: Fig. S1b**)**. Widespread inter-network coupling decreases were also observed in ALS-ES (all uncorrected *p* < 0.05), particularly between the SMN and the DAN, ventral attention network (VAN), and limbic network (LIM), as well as between the VAN and the frontoparietal network (FPN) and DMN (Fig. 2i, Additional file 1: Table S11) (See Additional file 1: Result S3 for details). After adjusting for the full set of six covariates, significant SMN decoupling remained consistent in both bulbar-onset (*F* = 5.546, *p*_*FDR*_ = 0.021) and limb-onset patients (*F* = 5.996, *p*_*FDR*_ =0.016) compared to HCs, with no significant difference between the two subgroups (*p* = 0.602). Furthermore, no significant correlation was observed between decoupling and disease duration (*r*=-0.169, *p* = 0.173), anxiety (HARS: *p* = 0.888), or depression scores (HDRS: *p* = 0.799). Sensitivity analyses accounting for affective symptoms further validated these findings. After adjusting for age, sex, TIV, head motion, HARS, and HDRS scores, significant SC-FC decoupling remained consistent in the SMN (*p*_*FDR*_ = 0.024) and key cortical areas, including the left precentral gyrus (*p*_*FDR*_ < 0.001). Detailed results for regional (Additional file 1: Table S12), intra-network (Additional file 1: Table S13), and inter-network (Additional file 1: Table S14) coupling under this full-variable control model are provided in the Additional file 1.

### Machine Learning Analysis

**Fig. 3:**
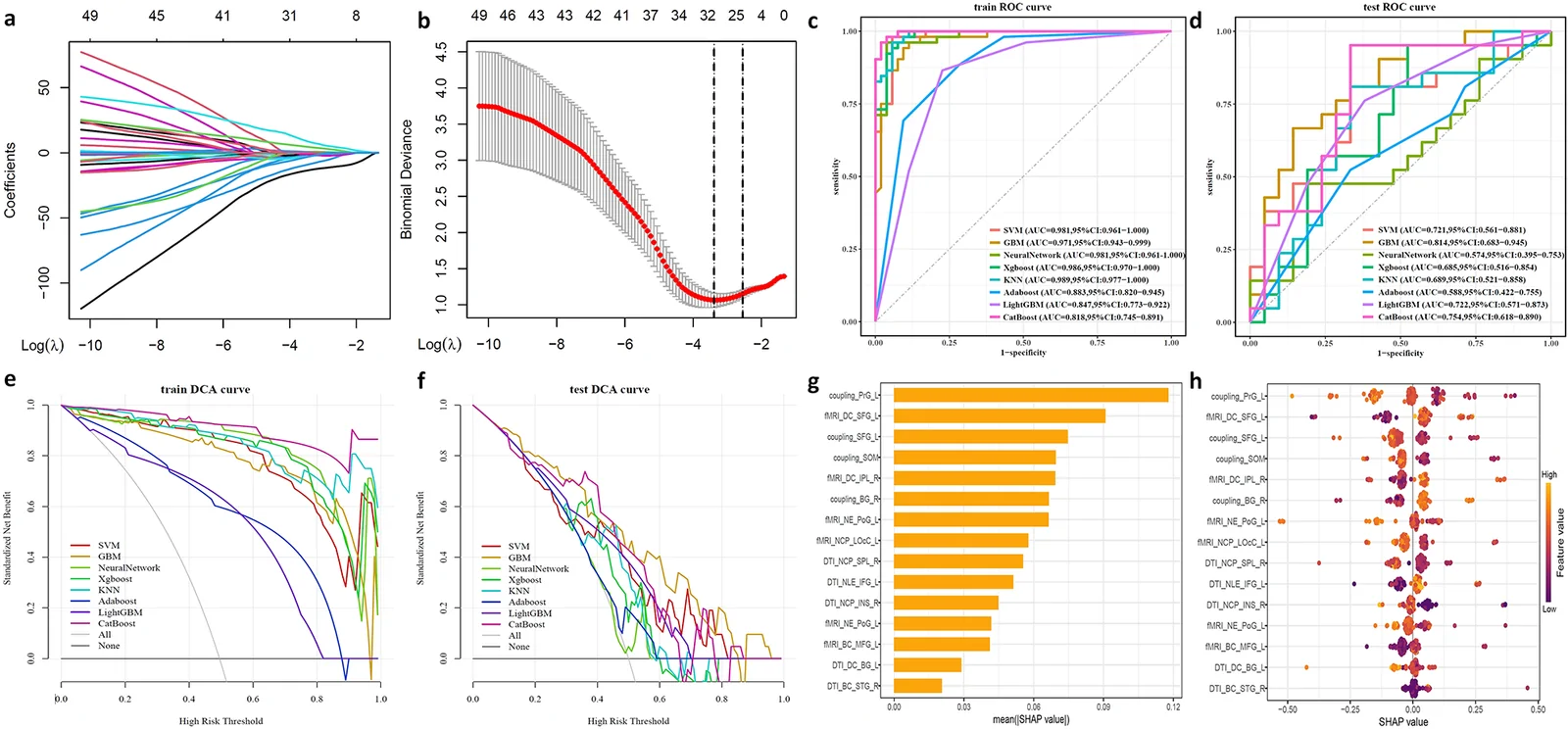
Machine Learning Model Selection, Performance, and Interpretation. **a** Ten-fold cross-validation for tuning the penalty parameter lambda in LASSO regression. **b** LASSO coefficient profiles showing feature selection process. **c** Training set Receiver Operating Characteristic (ROC) curves curves for all eight machine learning models. **d** Test set ROC curves evaluating model generalizability. **e** DCA assessing clinical utility on the training set. **f** DCA validating net clinical benefit on the test set. **g** SHAP-based feature importance ranking (top 15) in the GBM model. **h** SHAP summary plot illustrating feature value effects on model predictions. Abbreviations: SVM, Support Vector Machine; GBM, Gradient Boosting Machine; XGBoost, eXtreme Gradient Boosting; KNN, K-Nearest Neighbors; LightGBM, Light Gradient Boosting Machine; CatBoost, Categorical Boosting; AdaBoost, Adaptive Boosting; L, left; R, right; BC, betweenness centrality; DC, degree centrality; NCP, nodal clustering coefficient; NLE, nodal local efficiency; NE, nodal efficiency; STG, superior temporal gyrus; SPL, superior parietal lobule; PCL, paracentral lobule; pSTS, posterior superior temporal sulcus; BG, basal ganglia; IPL, inferior parietal lobule; INS, insula; LOcC, lateral occipital cortex; IFG, inferior frontal gyrus; MFG, middle frontal gyrus; PoG, postcentral gyrus; CG, cingulate gyrus; SFG, superior frontal gyrus; PrG, precentral gyrus; SMN, somatomotor network

To classify ALS-ES from HCs, a total of 3,302 multimodal features were initially extracted. After removing features with missing values, a final set of 3,036 features was utilized to build the machine learning pipeline. A two-stage feature selection, applied to the training set, first used a t-test to identify 76 features, which were then fed into a LASSO regression, yielding 29 optimal predictive features (Fig. 3a, b; Additional file 1: Table S15). Among the eight models trained and evaluated, the Gradient Boosting Machine (GBM) model achieved the highest test AUC (0.814) and demonstrated the best overall performance, with a test accuracy of 76.2% and an F1-score of 73.7% (Fig. 3c, d; Additional file 1: Fig. S2; Table S16). The robustness of this classification performance was further assessed using 1000 permutation tests, which generated a null distribution of AUC values by randomly shuffling group labels. The significance of the observed performance (*p*_*perm*_ < 0.001) confirms that the model’s predictive power significantly exceeds chance levels and is not driven by over-fitting (Additional file 1: Fig. S3a). Notably, the neuroimaging model substantially outperformed a clinical baseline model consisting of age and sex, which yielded a lower AUC of 0.644 (Additional file 1: Fig. S3b). DCA confirmed the GBM model’s superior clinical utility (Fig. 3e, f). SHAP analysis of the GBM model identified the top predictive features as: SC-FC coupling in the left PrG (coupling_PrG_L), functional MRI-based degree centrality in the left SFG (fMRI_DC_SFG_L), and SC-FC coupling in the left SFG (coupling_SFG_L) (Fig. 3g, h; Additional file 1: Fig. S4; Fig. S5). For clinical translation, we developed a web-based Shiny application that enables practical and individualized risk prediction (Additional file 1: Fig. S6) (See Additional file 1: Result S4 for details).

### Neurochemical and Cognitive Correlates of SC-FC Coupling

**Fig. 4:**
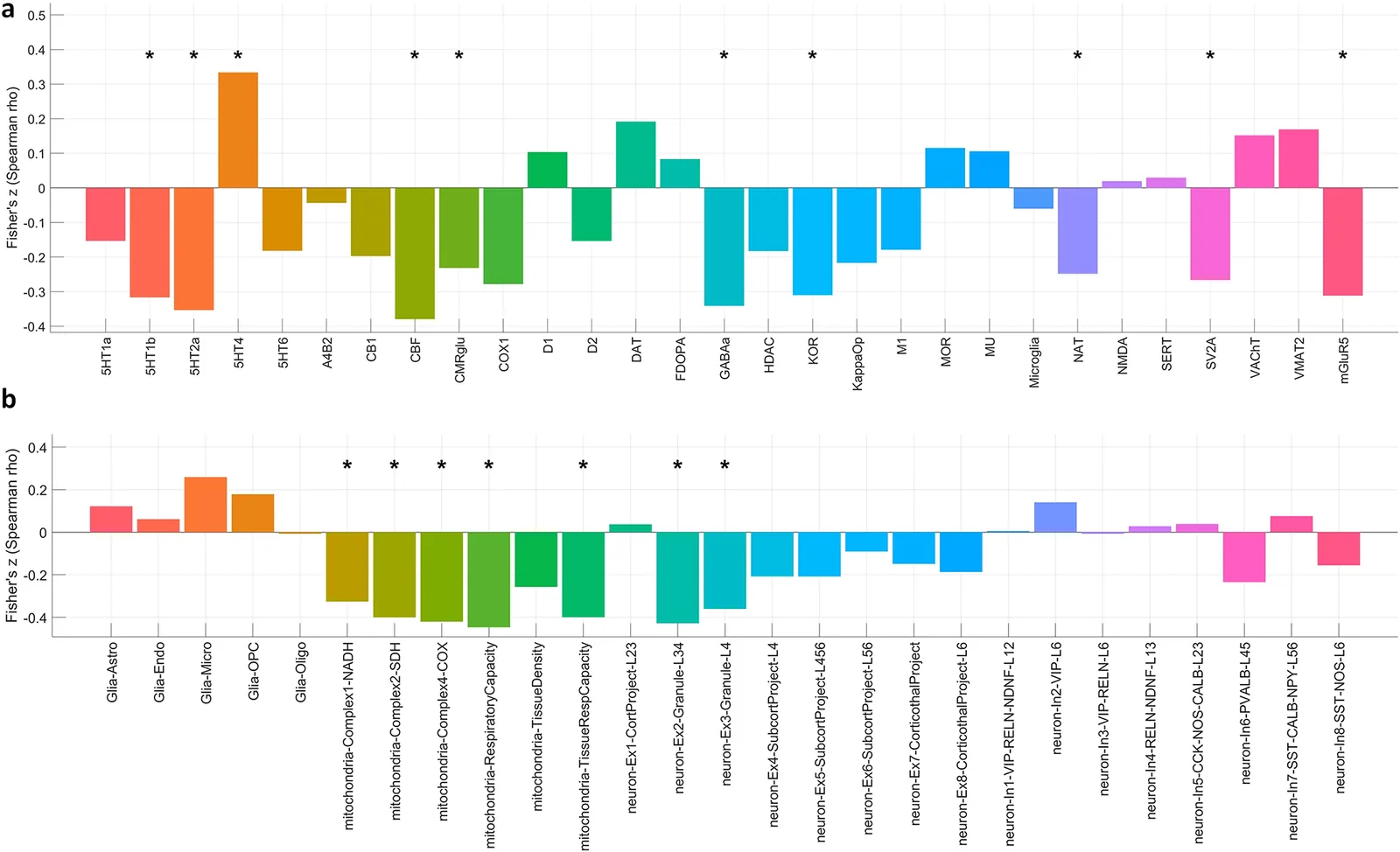
Neurochemical and cognitive correlates of SC-FC coupling alterations in ALS. **a** Correlation coefficients (r-values) between regional SC-FC coupling changes and neurotransmitter receptor/transporter distributions. **b** Correlations between SC-FC coupling changes and specific brain cell type markers. Asterisks indicate significant correlations surviving FDR correction (*p* < 0.05) Abbreviations: Astro, astrocyte; Endo, endothelial; Micro, microglia; OPC, oligodendrocyte precursor cell; Oligo, oligodendrocyte; NADH, nicotinamide adenine dinucleotide; SDH, succinate dehydrogenase; COx, cytochrome c oxidase; Ex, excitatory neuron; In/ln, inhibitory neuron; VIP, vasoactive intestinal polypeptide; RELN, reelin; NDNF, neuron-derived neurotrophic factor; CCK, cholecystokinin; NOS, nitric oxide synthase; CALB, calbindin; PVALB, parvalbumin; SST, somatostatin; NPY, neuropeptide Y; DMN, default mode network; PFC, prefrontal cortex; DMPFC, dorsomedial prefrontal cortex; MPFC, medial prefrontal cortex; M1, primary motor cortex; S1, primary somatosensory cortex; SII, secondary somatosensory cortex

Spatial correlation analysis identified significant associations between SC-FC coupling alterations and specific neurotransmitter systems. Significant negative correlations were observed with serotonin receptors 5-hydroxytryptamine 2 A (5-HT2A) (*r* = -0.35, *p*_FDR_ = 0.007), 5-hydroxytryptamine 1B (5-HT1B) (*r* = -0.32, *p*_FDR_ = 0.021), and metabotropic glutamate receptor 5 (mGluR5) (*r* = -0.31, *p*_FDR_ = 0.019), while a positive correlation was found for 5-hydroxytryptamine 4 (5-HT4) (*r* = 0.33, *p*_FDR_ = 0.007) (Fig. 4a). At the cellular level, SC-FC alterations showed strong negative correlations with mitochondrial complex markers (complex II: *r* = -0.40; complex IV: *r* = -0.42; respiratory capacity: *r* = -0.45; all *p*_FDR_ = 0.004) and excitatory neuron subtypes in layers 3–4 (*r* = -0.43, *p*_FDR_ = 0.004) (Fig. 4b). No significant correlations were detected for N-methyl-D-aspartate (NMDA), serotonin transporter (SERT), or major glial markers after multiple comparisons correction (Additional file 1: Tables S17, S18). To maintain a streamlined narrative focused on the core imaging-transcriptomic integration, these complementary neurochemical and cognitive mapping results are detailed in Additional file 1: Results S5 and Tables S19.

### Regional Gene Expression Pattern of SC-FC Coupling

**Fig. 5:**
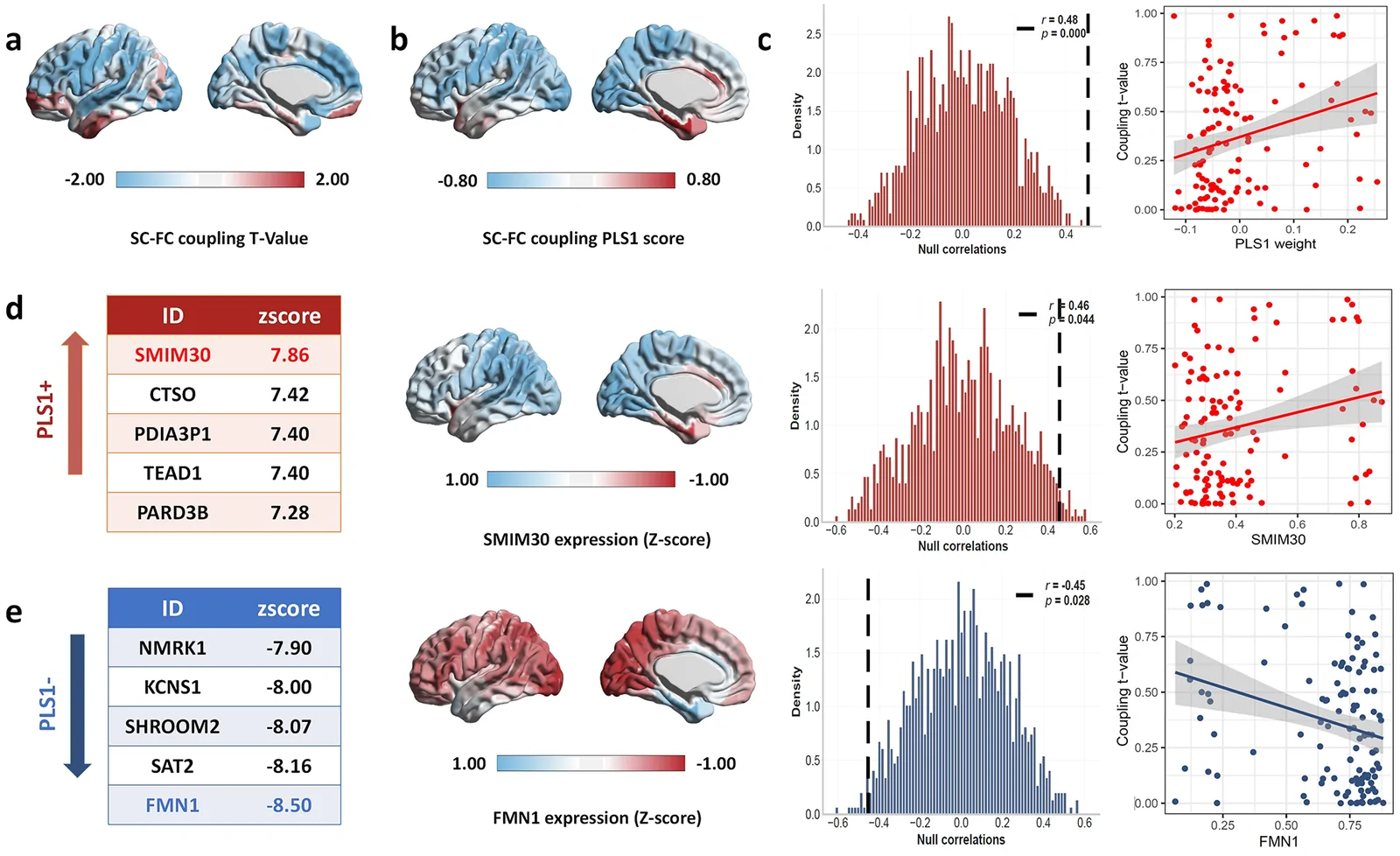
Gene expression profiles related to regional variation in SC-FC coupling.** a** Cortical distribution of SC-FC coupling in the left hemisphere (unthresholded). **b** Weighted gene expression map of regional PLS1 scores in the left hemisphere (unthresholded). **c** A scatterplot shows the relationship between regional PLS1 scores (weighted sum of 15,633 gene expression scores) and regional SC-FC coupling values (*r* = 0.56, *p*_spin_ < 0.05). **d** Genes strongly positively weighted on PLS1 (e.g., SMIM30) correlate positively with case-control differences in SC-FC coupling (*r* = 0.46, *p*_spin_ < 0.05). **e** Genes strongly negatively weighted on PLS1 (e.g., FMN1) correlate negatively with case-control differences in SC-FC coupling (*r* = -0.45, *p*_spin_ < 0.05)

We used PLS regression to identify the spatial pattern of gene expression that correlated with the anatomical distribution of the SC-FC coupling (Fig. 5a). The first PLS component (PLS1) explained the largest proportion of variance in SC-FC coupling (27.50%) and was significantly above chance (*p*_boot_ = 0.001). The distribution of the PLS1 weighted map reflected a robust anterior–posterior gene expression gradient (Fig. 5b). Notably, the PLS1 weighted gene expression map was spatially correlated with the case–control t-map of SC-FC coupling (Pearson’s *r* = 0.48, *p*_spin_ < 0.001) (Fig. 5c). To account for inherent spatial autocorrelation, the significance of this association was confirmed using 10,000 spatial permutation tests (spin tests). These identified gene signatures are interpreted as markers of baseline regional vulnerability rather than disease-induced molecular pathology. Ranking normalized PLS1 weights with univariate one-sample Z tests identified 357 positively weighted genes (PLS1+, *Z* > 4.65) and 628 negatively weighted genes (PLS1-, *Z* < -4.65), all surviving Bonferroni correction (*p*_Bonferroni_ < 0.05). The top positively weighted gene was Small Integral Membrane Protein 30 (SMIM30) (Fig. 5d), while the strongest negatively weighted gene was Formin 1 (FMN1) (Fig. 5e).

### Gene Set Enrichment Analysis

**Fig. 6:**
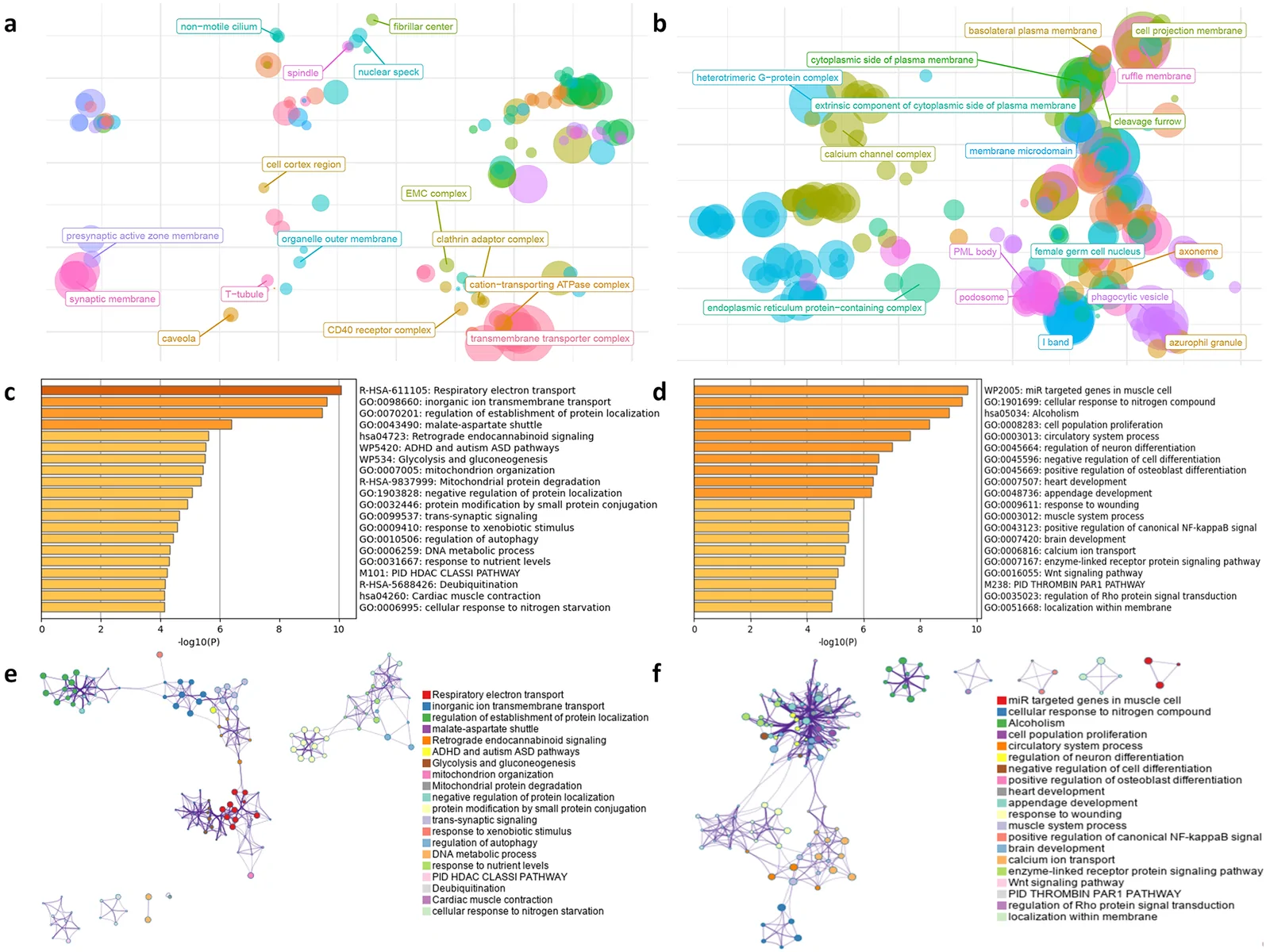
Functional Enrichment and Cellular Profiling of PLS1- and PLS1 + Genes. **a** GO enrichment analysis for PLS1- genes using rrvgo. **b** GO enrichment analysis for PLS1 + genes using rrvgo. **c** Bar plot of Metascape enrichment analysis for PLS1- genes. **d** Bar plot of Metascape enrichment analysis for PLS1 + genes. **e** Network visualization of enriched terms for PLS1- genes from Metascape. **f** Network visualization of enriched terms for PLS1 + genes from Metascape

To functionally characterize the genes associated with the first PLS component, we conducted gene set enrichment analysis on the PLS1- and PLS1 + gene sets. GO term analysis via clusterProfiler demonstrated a significant enrichment of PLS1- genes in biological processes implicated in ALS, such as transmembrane transport and ion channel activity, exemplified by the terms synaptic membrane and transmembrane transporter complex (Fig. 6a and Additional file 1: Fig. S7a, b). For PLS1 + genes, significant enrichment was observed for muscle system process and calcium ion transmembrane transport (Fig. 6b and Additional file 1: Fig. S7c, d). Further supporting this, gene set enrichment analysis using Metascape on the PLS1- gene list confirmed significant associations with ion channel-related pathways, including GO:0098660 (inorganic ion transmembrane transport) and GO:0099537 (trans-synaptic signaling) (Fig. 6c, e). Parallel analysis of the PLS1 + gene set with Metascape revealed significant enrichments for GO:0003012 (muscle system process) and GO:0006816 (calcium ion transport) (Fig. 6d, f). KEGG pathway analysis further supported these findings. PLS1- genes were enriched in cytoskeleton and cAMP signaling pathways (Additional file 1: Fig. S8a), while PLS1 + genes were notably enriched in NF-kB signaling and actin cytoskeleton regulation (Additional file 1: Fig. S8b) (See Additional file 1: Result S6 for details).

### Transcriptional Enrichment for Specific Cell Types and Cortical Layers

**Fig. 7:**
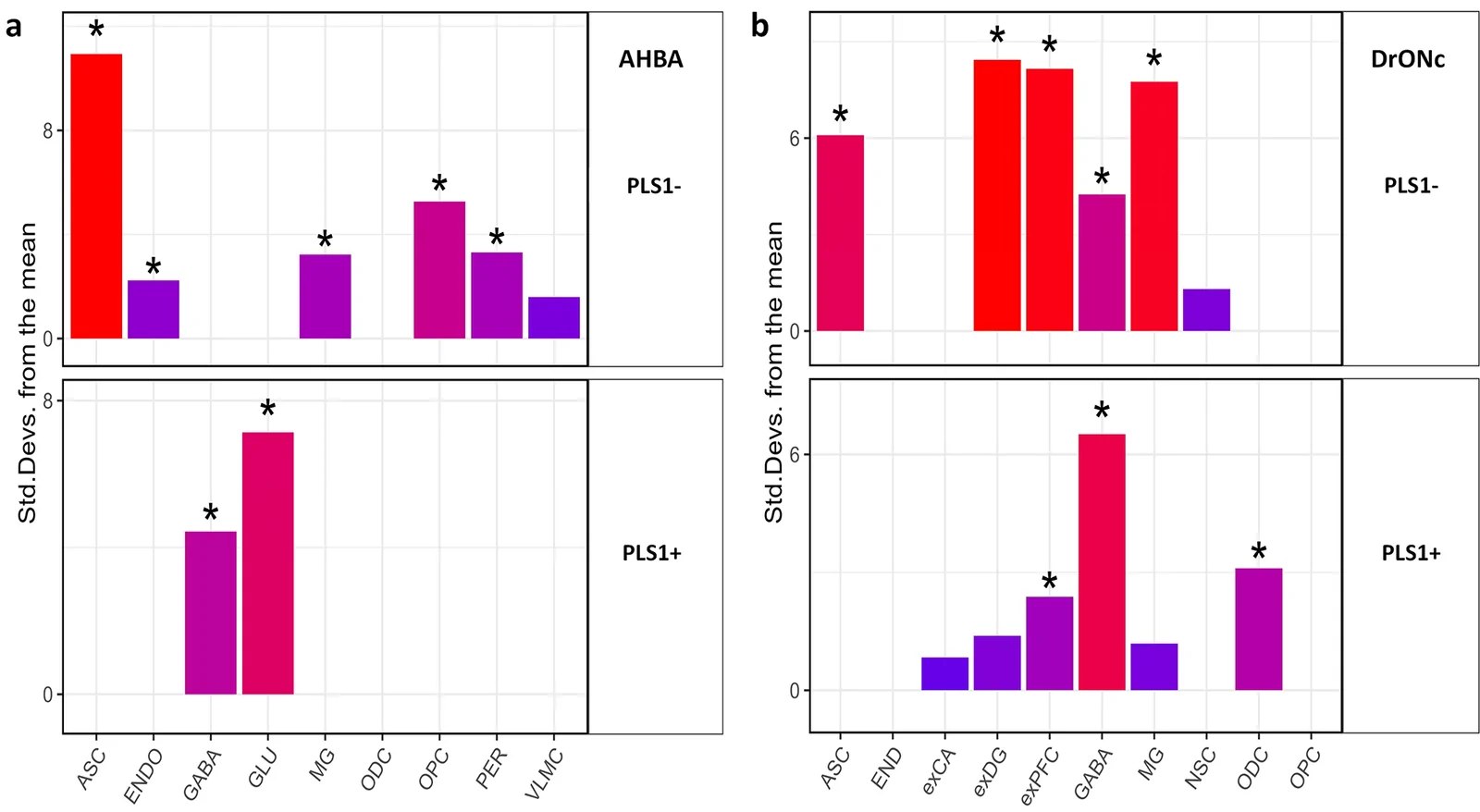
Cellular Profiling of PLS1- and PLS1 + Genes.**a** Expression enrichment of PLS1 + and PLS1- genes in AHBA-seq data. Bars are plotted only for FDR < 0.5; * indicates FDR < 0.05. **b** Expression enrichment of PLS1 + and PLS1- genes in DroNc-seq data, following the same conventions as in (g). Abbreviations: VLMC, vascular leptomeningeal cell; OPC, oligodendrocyte precursor cell; ODC, oligodendrocyte; MG, microglia; Inh_neuron, inhibitory neuron; Exc_neuron, excitatory neuron; ENDO, endothelial cell; ASC, astrocyte

Cell-type enrichment analysis was performed to assess whether the top 60% of PLS1 + and PLS1- genes associated with SC-FC coupling changes were preferentially expressed in specific brain cell types. Using AHBA-seq data, PLS1- genes were enriched in astrocytes (ASC), endothelial cells (ENDO), microglia (MG), oligodendrocyte precursor cell (OPC) and pericyte (PER), whereas PLS1 + genes were GABAergic neurons (GABA) and glutamatergic neurons (GLU) **(**Fig. 7a**)**. Consistent patterns were observed in DrONc-seq data: PLS1 + genes were expressed in glutamatergic neurons from the prefrontal cortex (exPFC), GABA and oligodendrocytes (ODC), while PLS1- genes were expressed in pyramidal neurons from the ASC, granule neurons from the hippocampal dentate gyrus region (exDG), exPFC, GABA and MG **(**Fig. 7b**)**. Across both datasets, GABA were the common enriched cell type for PLS1 + genes, whereas ASC and MG were consistently enriched for PLS1- genes. Cortical layers enrichment analysis further demonstrated that PLS1- genes were significantly enriched in layers III (NES = -2.34, *p* < 0.05) and layers V (NES = -1.77, *p* = 0.03) (Additional file 1: Fig. S9a, b). Conversely, the PLS1 + gene set showed no significant enrichment in any cortical layer (Additional file 1: Fig. S9c, d). The cortical layer distributions for the PLS1- and PLS1 + groups are detailed in Additional file 1: Table S20.

### Single-cell analysis

**Fig. 8:**
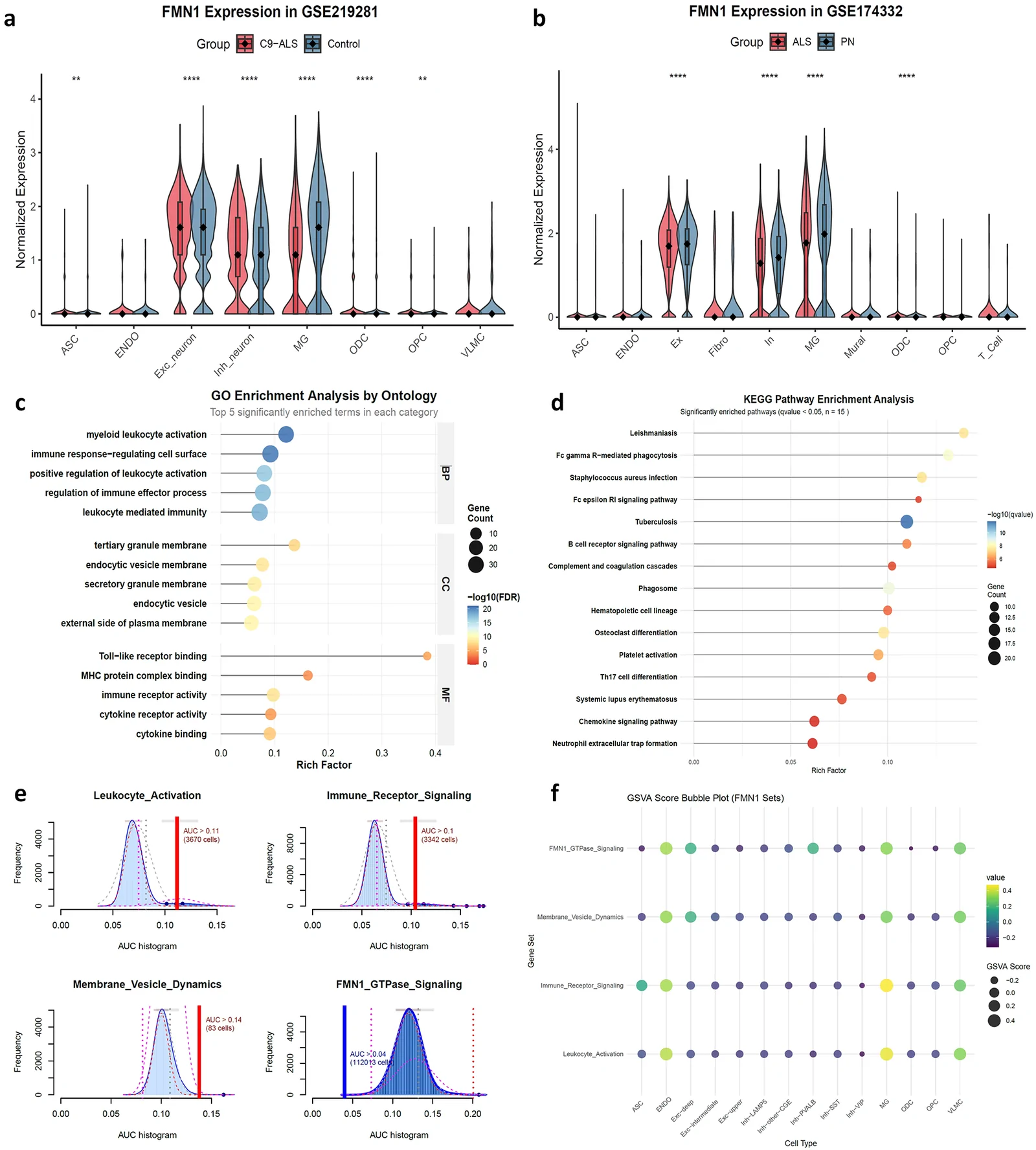
Single-cell analysis of FMN1 in ALS. **a** Violin plots comparing FMN1 expression between ALS patients and HCs in GSE219281. **b** Violin plots of FMN1 expression in ALS versus HCs (GSE174332). **c** GO and (**d**) KEGG enrichment analyses of microglial cells. (i) Volcano plot of differentially expressed genes in microglia (GSE219281), highlighting FMN1. **e** Cell assignment histogram based on FMN1-associated pathway module scores. **f** Gene Set Variation Analysis (GSVA) bubble plot showing enrichment of FMN1-related pathways across cell types

To explore the cellular basis of the PLS1 components, we analyzed genes with extreme PLS1 z-scores in dataset GSE219281. FMN1, the top PLS1- gene, was enriched in ASC and MG. FMN1 expression was significantly decreased in ASC and MG in GSE219281 (Fig. 8a; Additional file 1: Fig. S10a-c) and in MG in the validation dataset GSE174332 (Fig. 8b; Additional file 1: Fig. S10g-i), confirming a consistent reduction. Conversely, the top PLS1 + gene, SMIM30, showed enrichment in inhibitory neurons (inh_neuron). However, it yielded contradictory expression results between the GSE219281 (higher in ALS) (Additional file 1: Fig. S10d-f) and GSE174332 (lower in ALS) (Additional file 1: Fig. S10j-l) datasets and was excluded from further analysis. Subsequent GO/KEGG analysis of FMN1 in GSE219281 MG cells (Fig. 8c, d) revealed enrichment for immune and phagocytic pathways (Additional file 1: Table S21). To further investigate the functional impact of FMN1, we performed a comprehensive cell scoring analysis on four FMN1-associated pathways: “Leukocyte Activation”, “Immune Receptor Signaling”, “Membrane Vesicle Dynamics”, and “FMN1 GTPase Signaling”. Module scoring confirmed significant pathway alterations (*p* < 0.05) (Fig. 8e, f). Specifically, AUCell analysis was used to visualize the activity of these gene sets at the single-cell level, revealing distinct spatial enrichment patterns across the UMAP landscape (Additional file 1: Fig. S11a-d). Complementary violin plots quantitatively compared the AUCell score distributions, confirming that the activity of all four pathways was significantly different between the ALS and HCs groups (Additional file 1: Fig. S12a-d).

## Discussion

This study identified significant SC-FC decoupling in the premotor, frontal, and SMN of early-stage ALS patients. This metric was identified as the most discriminative feature within our machine learning framework. Spatially, this decoupling correlated negatively with 5-HT2A/mGluR5 receptor densities and mitochondrial function markers. Imaging-transcriptomics linked this network failure to a gene signature enriched for synaptic and ion channel pathways, preferentially expressed in microglia, and localized to cortical Layers III and V. Finally, single-cell analysis identified FMN1 as a candidate gene whose normative glial expression spatially overlaps with SC-FC decoupling. This topographic association provides exploratory evidence of regional molecular vulnerability potentially linked to macroscopic network failure in ALS.

A key finding of our study was the significant mismatch between brain structure and function in early-stage ALS. Specifically, while the macroscale anatomical framework remained largely stable, intra-network functional connectivity was significantly reduced within the SMN. This functional-structural mismatch manifested as a more pronounced and widespread alteration in SC-FC coupling than either modality alone. While nominal alterations appear widespread, the substantially larger effect size in the SMN identifies it as the primary epicenter of network failure in early-stage ALS. Indeed, our findings reveal significant alterations in SC-FC coupling in ALS patients at both regional and network levels, consistent with the neurodegenerative nature of the disease [8]. Regionally, the greatest SC-FC decline was concentrated in the motor cortices and prefrontal areas. While advanced techniques like probabilistic tractography or neurite orientation and dispersion density imaging (NODDI) often reveal earlier microstructural changes [37, 38], our results demonstrate that functional coordination fails even when the macroscale anatomical architecture remains statistically stable. While prior studies reported early white matter damage in the corticospinal tract [39, 40], our structural networks remained stable. This discrepancy reflects our conservative deterministic tractography and FN-based weighting, which prioritize anatomical stability for coupling analysis but offer lower sensitivity to microstructural loss than probabilistic methods. Crucially, significant SC-FC decoupling emerged despite this macroscale stability, suggesting that functional coordination fails before gross structural connectivity loss in early-stage ALS. Decoupling in the motor cortex directly supports ALS as a motor neuron disease, indicating that structural damage in the disease’s core regions compromises their functional integration [41]. This is consistent with evidence showing the paracentral lobule and precentral gyrus are among the most structurally and functionally impaired nodes [42]. Prefrontal involvement aligns with the view that ALS is a widespread brain network disorder, reflecting early encroachment into non-motor cognitive areas [43]. Crucially, we observed significant inter-network decoupling between the SMN and several other systems, including the DAN, VAN, and LIM [13]. This suggests ALS is not confined to the motor system, but impairs the flow and integration of information across networks governing movement, attention, and emotion. This broad functional imbalance may underlie the diverse cognitive, behavioral, and motor symptoms experienced by ALS patients.

We observed significant negative correlations between SC-FC alterations and the distributions of 5-HT2A, 5-HT1B, and mGluR5 receptors. Rather than implying direct receptor loss, this topographic alignment suggests that the serotonin pathway may represent a potential biological landscape associated with regional vulnerability in ALS. These results highlight a spatial overlap between systemic network failure and the underlying normative receptor architecture, providing exploratory evidence of neurochemical landscapes that may be linked to disease susceptibility [44, 45]. Similarly, the spatial decoupling from the mGluR5 receptor, central to synaptic plasticity and glutamate-mediated excitotoxicity [46], may reflect a failure of neuronal compensation or receptor loss [47], contributing to the collapse of network integration. Furthermore, SC-FC changes showed a significant negative correlation with markers for mitochondrial complexes and respiratory capacity. This strongly links SC-FC anomalies to cellular energy deficits, a hallmark pathology of ALS [48]. Cognitive analysis revealed that the most decoupled regions were precisely those associated with sensory processing, motor coordination, and medial prefrontal cortex functions [13]. This provides key neuroimaging evidence that SC-FC decoupling localizes to functional regions underlying both core motor and frontal non-motor symptoms in ALS.

Our study employed PLS regression to link the spatial pattern of macroscopic SC-FC coupling alterations with regional gene expression from the AHBA, revealing key molecular mechanisms of structural-functional breakdown. The gene set negatively correlated with SC-FC changes (PLS1-) was highly enriched in biological processes such as ion channel activity, transmembrane transport, and synaptic transmission [49]. Low expression of these genes, which represent a core functional module for maintaining synaptic homeostasis [42], tends to coincide with lower SC-FC coupling. Dysfunction or downregulation of genes involved in ion channel function and synaptic vesicle cycling [50] likely leads directly to neuronal and synaptic dysfunction [51]. This weakens the support provided by structural white matter tracts for functional activity, manifesting as reduced SC-FC coupling. This mechanism aligns closely with the extensively reported synaptic dysfunction and excitation/inhibition imbalance in ALS [52]. Conversely, the gene set positively correlated with SC-FC changes (PLS1+) was enriched in processes related to calcium ion transport and the muscular system. High expression of these genes often accompanied stronger SC-FC coupling, potentially representing a compensatory or protective signature. For instance, the enrichment of PLS1 + genes in GABAergic neurons may represent a molecular-level adaptive response aimed at maintaining network stability during early-stage network failure. Similarly, the upregulation of calcium transport genes might reflect a neural response to manage intracellular calcium homeostasis [53]. Alternatively, this signal could indicate the concurrent activation of neuroinflammatory or cellular stress pathways [54]. Given that ALS pathology includes motor neuron degeneration and early neuromuscular junction disruption, gene changes related to muscular processes [55], even in cortical regions, may reflect the central nervous system’s adaptive feedback or regulation in response to peripheral neuro-muscular decline. This multiscale association, identified via spatial permutation and abagen-standardized mapping, highlights baseline regional vulnerabilities that potentially predispose the motor system to decoupling.

Integrating macroscale connectomics with microscale cellular and laminar gene expression allowed us to pinpoint the pathological origin of SC-FC coupling changes in ALS. The negatively correlated gene set (PLS1–) was enriched in ASC and MG. The astrocytic enrichment suggests that compromised supportive functions, particularly in glutamate clearance and ion homeostasis, may be closely associated with the observed SC-FC decoupling [56]. MG, the resident macrophages of the CNS parenchyma, are strongly associated with the complex neuroinflammatory process in ALS [57]. MG support neuronal homeostasis, for instance, by secreting cytokines and chemokines, pruning synapses, and phagocytosing dead cells [58]. We hypothesize that the observed impairment in SC-FC coupling stems from a failure of this microglial regulation. Conversely, the positively correlated gene set (PLS1+) was enriched in GABAergic neurons. Enrichment in GABAergic neurons points to inhibitory neuronal dysfunction as a key molecular basis for reduced SC-FC coupling. Given that early ALS involves reduced cortical inhibition and excitation-inhibition balance, the low expression of PLS1 + genes in GABAergic neurons likely reflects this inhibitory deficit at the transcriptomic level, impacting functional integration [59]. Our study revealed significant enrichment of PLS1- genes in cortical layers III and V. Layer V houses corticospinal motor neurons forming the crucial motor output pathway, whose degeneration with impaired glutamate transport represents a core ALS pathology [60, 61]. Concurrently, layer III involvement corresponds to cognitive impairment-related changes observed in frontotemporal regions [62]. Thus, the laminar distribution of PLS1- genes indicates that SC-FC decoupling reflects combined damage to both motor output pathways and intracortical processing circuits.

Our single-cell analysis also revealed a significant downregulation in microglia, a cell type identified in our enrichment for specific cell types and which is critical in ALS pathology [57]. Microglia play a dual, complex role in neurodegeneration, capable of both neuroprotection and neurotoxicity, often by mediating synaptic pruning and inflammation [58, 63]. FMN1 (Formin 1) is a gene encoding a protein of the formin family. Its main function is to regulate the dynamic reorganization of the actin cytoskeleton, including actin nucleation, bundling, and microtubule organization [64]. We hypothesize that in microglia, FMN1 may be involved in SC-FC coupling by affecting cytoskeleton remodeling, participating in critical processes such as immune response, phagocytosis, and cell migration. To explore this, we performed pathway enrichment analysis within the microglial cell cluster and FMN1-related pathways, which identified significant alterations in pathways such as ‘Leukocyte Activation‘ [65], ‘Immune Receptor Signaling‘ [66], ‘Membrane Vesicle Dynamics‘ [67], and ‘FMN1 GTPase Signaling‘ [68]. We then quantified the activity of these specific pathways using module scoring. This confirmed that the activity of these microglial-associated immune and phagocytic pathways was significantly altered in ALS. This transcriptomic shift may be reflect by a decline in microglial phagocytic capacity, leading to an inability to effectively clear perisynaptic pathological proteins [67], and an increase in the secretion of pro-inflammatory factors, exacerbating neuroinflammation [65]. This widespread cellular-level dysfunction likely manifests as the observed macroscopic decoupling between structural connectivity and functional activity. While microglial activation is common in neurodegeneration, our findings support a degree of ALS-specificity because the identified gene signatures were enriched in microglia and FMN1 downregulation was validated in independent ALS single-cell datasets. However, these associations remain spatial correlates and do not establish a direct causal link between microglial FMN1 expression and macroscale network failure.

This study has several limitations. First, transcriptomic associations derived from healthy AHBA donor data, which primarily sample the left hemisphere, may reflect baseline regional vulnerability rather than disease-induced molecular pathology. While we utilized abagen and spin tests to control for sampling density and spatial autocorrelation, these patterns might still partly overlap with generic cortical organization. Second, the cross-sectional design precludes definitive causal inferences regarding disease initiation. Consequently, whether SMN decoupling acts as a primary trigger or a secondary consequence remains speculative without longitudinal follow-up data. Third, features optimized for classification performance are not inherently primary biological drivers. While our findings highlight SC-FC decoupling as a sensitive predictive signature, these results should be viewed as statistical candidates requiring further multi-scale validation to confirm their pathological significance. Furthermore, the link between microglial FMN1 expression and macroscopic coupling is currently indirect, necessitating future experimental studies to confirm its functional role in ALS-related network failure. Future studies should incorporate longitudinal designs, disease-specific transcriptomics, and independent multi center cohorts to validate these findings.

## Conclusions

In conclusion, our multimodal analysis demonstrates that early ALS is associated with by SC-FC decoupling in the SMN, a key feature in our machine learning classifier. This network dysfunction correlates with reduced 5-HT2A/mGluR5 receptor density and mitochondrial impairment. We traced this to a synaptic and ion channel gene signature enriched in microglia, with FMN1 emerging as a top candidate gene in independent single cell validation. This multiscale work bridges macroscopic network dysfunction with specific molecular-cellular pathology, although longitudinal studies are needed to confirm the causal trajectories.

## Supplementary Information

Below is the link to the electronic supplementary material.

Supplementary Material 1. Additional File 1: Supplementary Methods, Results, Figures S1-S12, and Tables S1-S21. Supplementary Methods S1-S11: Detailed specifications for MRI acquisition, structural and functional brain network construction, SC-FC coupling analytical specifications, machine learning pipelines (feature selection, modeling, and interpretability), imaging-transcriptomics processing, and single-cell RNA-seq analysis. Supplementary Results S1-S8: Detailed statistical comparisons of global and nodal network properties, modularity analysis, regional SC-FC coupling alterations, machine learning model performance evaluation, and comprehensive bioinformatics/single-cell enrichment results. Supplementary Figures S1-S12: Including effect size of SC-FC decoupling (Fig. S1), confusion matrices (Fig. S2), model validation (Fig. S3), feature importance (Fig. S4), SHAP interpretability analysis (Fig. S5), web-tool interface (Fig. S6), GO/KEGG enrichment circular and bar plots (Fig. S7-S8), GSEA laminar profiles (Fig. S9), single-cell expression of FMN1 and SMIM30 (Fig. S10), and AUCell activity maps (Fig. S11-S12). Supplementary Tables S1-S21: Comprehensive data tables for global/nodal metrics, inter-network connectivity, LASSO coefficients, machine learning performance metrics, neurotransmitter/cognitive correlations, and pathway enrichment details.

## Data Availability

The data supporting the findings of this study are included in the article and its Additional file 1. Publicly available datasets were sourced from the following repositories: neurotransmitter receptor and transporter data from the JuSpace toolbox (https://github.com/juryxy/JuSpace/); and regional brain atlas information from the Brainnetome Atlas 246 (https://atlas.brainnetome.org/). Motor cortex transcriptomic data were obtained from the Gene Expression Omnibus (GEO) under accession numbers GSE219281 (https://identifiers.org/geo: GSE219281) and GSE174332 (https://identifiers.org/geo: GSE174332). Transcriptomic decoding and functional enrichment analyses were conducted using Metascape (https://metascape.org). Additional data generated in this study are available from the corresponding author upon reasonable request.

## Abbreviations

ALS: Amyotrophic lateral sclerosis
ALS-ES: Early stage amyotrophic lateral sclerosis
HCs: Healthy controls
SC: Structural connectivity
FC: Functional connectivity
SC-FC: Structural-functional coupling
VIS: Visual network
VAN: Ventral attention network
FPN: Frontoparietal network
LIM: Limbic network
DMN: Default mode network
DAN: Dorsal attention network
SMN: Somatomotor network
SVM: Support Vector Machine
GBM: Gradient Boosting Machine
XGBoost: eXtreme Gradient Boosting
KNN: K-Nearest Neighbors
LightGBM: Light Gradient Boosting Machine
CatBoost: Categorical Boosting
AdaBoost: Adaptive Boosting
BC: Betweenness centrality
DC: Degree centrality
NCP: Nodal clustering coefficient
NLE: Nodal local efficiency
NE: Nodal efficiency
VLMC: Vascular leptomeningeal cell
OPC: Oligodendrocyte precursor cell
ODC: Oligodendrocyte
MG: Microglia
Inh_neuron: Inhibitory neuron
Exc_neuron: Excitatory neuron
ENDO: Endothelial cell
ASC: Astrocyte
AHBA: Allen Human Brain Atlas
GEO: Gene Expression Omnibus
PLS: Partial Least Squares
GO: Gene Ontology
KEGG: Kyoto Encyclopedia of Genes and Genomes
EWCE: Expression Weighted Cell-type Enrichment
ssGSEA: Single-sample Gene Set Enrichment Analysis
UMAP: Uniform Manifold Approximation and Projection
GSVA: Gene Set Variation Analysis
ALSFRS-R: Amyotrophic Lateral Sclerosis Functional Rating Scale-Revised
HARS: Hamilton Anxiety Rating Scale
HDRS: Hamilton Depression Rating Scale
FDR: False Discovery Rate
ROC: Receiver Operating Characteristic
DCA: Decision Curve Analysis
SHAP: SHapley Additive exPlanations
LASSO: Least Absolute Shrinkage and Selection Operator
