# Supplementary material for: Structural-functional network decoupling in early stage amyotrophic lateral sclerosis reveals cell-type specific transcriptional signatures
Source: BMC Med. 2026 Jul 2;24:511. doi: 10.1186/s12916-026-04932-7 (PMC13599184; doi:10.1186/s12916-026-04932-7)
Supplement: Supplementary file 1 [file 12916_2026_4932_MOESM1_ESM.docx]

**Structural-functional network decoupling in early stage amyotrophic lateral sclerosis reveals cell-type specific transcriptional signatures**

**CONTENT**

[Methods	1](#_Toc32086)

[S1 Detailed MRI Acquisition Parameters	1](#_Toc22885)

[S2 Structural brain network construction and analysis	1](#_Toc19279)

[S3 Functional brain network construction and analysis	3](#_Toc23228)

[S4 SC-FC Coupling Analytical Specifications	4](#_Toc22182)

[S5 Machine Learning Feature Selection	5](#_Toc5959)

[S6 Machine Learning Algorithm Modeling and Evaluation	6](#_Toc24438)

[S7 Model Interpretability Analysis	7](#_Toc13096)

[S8 Neurotransmitter and Cognitive Analysis Details	7](#_Toc5120)

[S9 Imaging-Transcriptomics Processing Pipeline	8](#_Toc31645)

[S10 Bioinformatics and Enrichment Analysis	9](#_Toc3212)

[S11 Single-Cell Analysis	10](#_Toc4065)

[Results	11](#_Toc31530)

[S1 Structural Network Analysis	11](#_Toc21090)

[S1.1 Global Topological Properties	11](#_Toc22192)

[S1.2 Nodal Topological Properties	11](#_Toc27735)

[S1.3 Intra-Network Modularity Analysis	14](#_Toc16994)

[S1.4 Inter-Network Modularity Analysis	14](#_Toc16175)

[S2 Functional Network Analysis	15](#_Toc16427)

[S2.1 Global Topological Properties	16](#_Toc14978)

[S2.2 Nodal Topological Properties	16](#_Toc26959)

[S2.3 Intra-Network Modularity Analysis	17](#_Toc605)

[S2.4 Inter-Network Modularity Analysis	18](#_Toc2560)

[S3 SC-FC coupling Analysis	19](#_Toc28965)

[S3.1 Global and Regional SC-FC Coupling	19](#_Toc7736)

[S3.2 Intra- and Inter-Network SC-FC Coupling	20](#_Toc6506)

[S3.3 Sensitivity Analysis of Structural-Functional Coupling	22](#_Toc25285)

[S4 Machine Learning Analysis	25](#_Toc20308)

[S4.1 Feature Selection	25](#_Toc884)

[S4.2 Model Construction and Evaluation	27](#_Toc16029)

[S4.3 Model Interpretability Analysis	29](#_Toc30642)

[S5 Neurochemical and Cognitive Correlates of SC-FC Coupling	31](#_Toc31028)

[S6 Gene set enrichment analysis	35](#_Toc26914)

[S7 Enrichment for specific cell types and cortical layers	36](#_Toc3053)

[S8 Single-cell analysis	37](#_Toc7160)

[S8.1 Gene Expression Analysis	37](#_Toc20994)

[S8.2 GO and KEGG Pathway Analysis	40](#_Toc20250)

[S8.3 Cell Scoring Analysis	41](#_Toc17696)

# Methods

S1 Detailed MRI Acquisition Parameters
All magnetic resonance imaging (MRI) data were acquired using a 3.0T Philips Ingenia scanner equipped with a dStream head coil. High-resolution T1-weighted images were obtained using a three-dimensional (3D) fast spoiled gradient-recalled echo (FSPGR) sequence with the following parameters: repetition time (TR) = 6.7 ms, echo time (TE) = 3.0 ms, matrix size = 240×240×170, voxel size = 1 mm³ isotropic, field of view (FOV) = 240×240 mm, 180 slices. Resting-state functional magnetic resonance imaging (rs-fMRI) data were acquired using an echo-planar imaging (EPI) sequence: TR = 2000 ms, TE = 30 ms, FOV = 230×230×144 mm, matrix = 68×68, voxel size = 3.5×3.5×4 mm, slice thickness = 4 mm, no slice gap, 32 slices, 240 volumes. Diffusion-Tensor images (DTI) were collected using a spin-echo echo-planar imaging (SE-EPI) sequence: TR = 4900 ms, TE = 95 ms, FOV = 224×224 mm, matrix = 112×112, b-values = 0 and 1000 s/mm², voxel size = 2 mm³ isotropic, 32 diffusion directions, 70 slices. Total intracranial volume (TIV) was estimated using the FreeSurfer 6.0 software suite (http://surfer.nmr.mgh.harvard.edu/). The estimated TIV was derived for each participant through the standard "recon-all" processing pipeline, which utilizes the relationship between the intracranial volume and the linear transform to MNI305 space.

## S2 Structural brain network construction and analysis

Network nodes were defined using the Brainnetome Atlas-246 (BNA-246), which provides fine-grained functional and anatomical parcellations for mapping brain connectivity. For each participant, T1-weighted images were first linearly co-registered to the diffusion space b0 image, then normalized to the Montreal Neurological Institute (MNI) T1 template. The BNA-246 atlas in MNI space was subsequently inversely transformed into individual diffusion space using nearest-neighbor interpolation to preserve discrete label values. The edge weight between nodes was defined as the product of fractional anisotropy (FA) and fiber number (FN). To minimize false-positive connections from the FACT tractography algorithm, an FN threshold of 3 was applied; only stream counts ≥3 were retained. Additionally, a group-level consistency threshold was used: a connection was considered valid only if it was present in at least 75% of participants. This yielded a 246 × 246 FA-weighted structural connectivity (SC) matrix for each subject. Graph theoretical analysis was performed using the Graph Theoretical Network Analysis (GRETNA) toolbox. Global network properties were computed to assess network integration and segregation, including clustering coefficient (Cp), characteristic path length (Lp), normalized Cp (Gamma), normalized Lp (Lambda), small-worldness (Sigma), global and local efficiency (Eglob, Eloc), and hierarchy. Nodal metrics, such as degree centrality (DC), betweenness centrality (BC), nodal efficiency (Ne), nodal local efficiency (NLe), nodal clustering coefficient (NCp), and nodal shortest path length (NLp), were used to evaluate the topological importance of individual brain regions. The BNA-246 regions were grouped into seven Yeo-based functional networks: visual network (VIS), somatomotor network (SMN), dorsal attention network (DAN), ventral attention network (VAN), limbic network (LIM), frontoparietal network (FPN), and default mode network (DMN). Mean intra-network and inter-network structural connectivity values were calculated for each subject. All statistical models adjusted for age, sex and intracranial total volume (TIV) as covariates. Significance was determined using a false discovery rate (FDR) corrected threshold of *p_FDR_* < 0.05.

S3 Functional brain network construction and analysis
For whole-brain functional connectivity (FC) analysis, preprocessing of resting-state fMRI data was performed using the GRETNA toolbox in MATLAB 2013b. The steps included: removing the first 10 time points, applying slice timing correction, and performing head motion correction. Subjects with head motion exceeding 3.0 mm translation or 3.0°rotation were excluded. Individual T1-weighted structural images were co-registered to the mean functional image, followed by spatial normalization to MNI space using the Diffeomorphic Anatomical Registration Through Exponentiated Lie algebra (DARTEL) algorithm with resampling to 3 mm isotropic voxels. Linear drift removal, regression of nuisance covariates (including Friston 24 head motion parameters, white matter, and cerebrospinal fluid signals), and band-pass filtering (0.01–0.10 Hz) were applied to reduce physiological noise. Based on the BNA-246 atlas, the brain was parcellated into 246 regions of interest (ROIs) to construct functional connectivity networks. The mean time series of each ROI was extracted, and Pearson correlation coefficients between all ROI pairs were computed to represent FC strengths. To control for individual differences and minimize the impact of arbitrary threshold selection, a network sparsity (S) thresholding approach was adopted, defined as the ratio of retained connections to the maximum possible connections. The sparsity range was set from 0.05 to 0.50, determined based on maintaining network connectedness and small-world properties (Sigma > 1.1) across all subjects. The area under the curve (AUC) for each topological metric over this sparsity range (in steps of 0.01) was calculated as a integrated measure to characterize network properties. Binary adjacency matrices were generated by retaining positive correlations within the sparsity range. Global and local topological properties were computed using GRETNA, consistent with the methodology applied in structural network analysis. Modularity analysis was conducted to assess the organization of densely interconnected brain regions. The brain was partitioned into seven functional subnetworks according to the Yeo template. The AUC values of intra-network and inter-network functional connectivity across the sparsity range were calculated as summary metrics for subsequent analysis. All statistical models adjusted for age, sex and TIV as covariates. Significance was determined using a FDR corrected threshold of *p_FDR_* < 0.05.

S4 SC-FC Coupling Analytical Specifications
SC-FC coupling analysis refers to the correlation between structural connectivity and its functional counterpart as described in prior research. This relationship was investigated by calculating correlation coefficients at three distinct levels. (1) At the whole-brain connection level, coupling non-zero SC values from each subject's structural network matrix were extracted into a vector. Corresponding FC values from the functional network matrix were also formed into a vector. SC-FC coupling was quantified as the Spearman correlation coefficient between these two vectors representing all connections across the entire brain. This produced one global SC-FC coupling value per subject reflecting overall structure-function consistency. (2) At the regional level, coupling the functional network was sparsified by setting connections without a corresponding structural link to zero. For each node, non-zero values from its corresponding row in both the SC and FC matrices were extracted into vectors. The spearman correlation between these vectors defined the nodal SC-FC coupling value. This process transformed each subject's 246×246 matrix into a 246×1 vector of regional coupling values indicating local structure-function consistency for each brain region. (3) At the subnetwork level, coupling analysis focused on intra-network and inter-network coupling. Intra-network coupling measured the correlation of structural and functional connection strengths among all region of interest pairs within the same Yeo-defined subnetwork. Inter-network coupling measured this correlation for connections linking a subnetwork's regions to all regions in other subnetworks. Thus, these correlations defined the intra-network and inter-network SC-FC coupling values, thereby quantifying the degree of functional segregation and integration within and across brain networks, respectively. All statistical models adjusted for age, sex and TIV as covariates. Significance was determined using a FDR corrected threshold of *p_FDR_* < 0.05.

## S5 Machine Learning Feature Selection

Features derived from DTI structural network analysis, fMRI functional network analysis, and structural-functional coupling analysis underwent a selection process. As the number of features substantially exceeded the sample size, feature selection was a necessary preliminary step before training the machine learning models. This was done to remove redundant predictors and thereby enhance generalization performance through feature subset selection and dimensionality reduction. This study employed a two-stage feature selection strategy that combined filter and embedded methods. To ensure the integrity of performance estimates and prevent data leakage, all group-dependent procedures, including data normalization and feature selection, were strictly nested within each cross-validation fold. Specifically, a t-test was applied independently to the training set of each fold as a primary analysis to filter out imaging features with a p-value greater than 0.01 Subsequently, the least absolute shrinkage and selection operator (LASSO) was used on the filtered training data for further feature screening. LASSO regression adds a penalty term, based on the sum of the absolute values of the coefficients, to the traditional linear regression loss function. By adjusting a regularization parameter to control the penalty's intensity, it achieves both coefficient shrinkage and automatic feature selection. This process promotes model sparsity by driving some coefficients towards zero and automatically eliminates features with minimal contribution to prediction, thereby retaining the most discriminative features for model performance. Crucially, normalization parameters and feature subsets were derived exclusively from the training set and then applied to the corresponding test set within each fold, ensuring that no information from the test set influenced the model training phase.

## S6 Machine Learning Algorithm Modeling and Evaluation

This study utilized eight machine learning classification algorithms to investigate their suitability and effectiveness. The selected algorithms were support vector machine (SVM), gradient boosting machine (GBM), neural network, extreme gradient boosting (XgBoost), K-nearest neighbors (KNN), adaptive boosting (AdaBoost), light gradient boosting machine (LightGBM), and categorical boosting (CatBoost). For hyperparameter selection, ten-fold cross-validation was combined with a random search method to identify the optimal combination of hyperparameters for each model. This procedure involved evaluating the predictive performance of every hyperparameter combination on the training set to progressively refine and optimize the selection range. Based on the optimal hyperparameters, the selected models were then trained using the training set data to ensure maximum predictive capability. The discriminative efficacy of the models was evaluated by constructing receiver operating characteristic (ROC) curves and calculating the area under the curve (AUC). The ROC curve provides a visual representation of a classifier's performance by plotting the true positive rate against the false positive rate at various classification thresholds. Additional evaluation metrics, including accuracy, sensitivity, specificity, precision, and F1-score, were also incorporated. These metrics are widely used in machine learning and assess model classification effectiveness from multiple dimensions. To ensure the robustness of the results, a bootstrapping method with 1000 repetitions was used to calculate the 95% confidence interval for the AUC. Furthermore, decision curve analysis (DCA) was introduced to evaluate the potential clinical utility of the models. This method quantifies the net benefit at different threshold probabilities to provide a scientific basis for clinical decision-making.

## S7 Model Interpretability Analysis

A performance comparison of the models constructed from the eight machine learning algorithms was conducted, and the best-performing algorithm was selected as the final predictive model. To improve the interpretability of this model, the SHAP (SHapley Additive exPlanations) method was utilized for analysis. Shapley values fairly distribute the contribution to the prediction outcome among the features, quantifying the impact of each feature on the final result and explaining how these features influence the model's output. By applying this explainable machine learning technique, the key features affecting the predictions and the process of their influence are visualized. This enhances the model's transparency and interpretability, providing strong support for more reliable decision-making. All statistical analyses were conducted using R version 4.1.0. All statistical tests were two-sided, and a *p* < 0.05 was considered statistically significant.

S8 Neurotransmitter and Cognitive Analysis Details
The JuSpace toolbox was employed to examine whether the spatial patterns of SC-FC coupling alterations correlated with canonical neurotransmitter distributions derived from nuclear imaging data. Spearman correlations were computed across all regions of the neuromorphometrics atlas. Permutation testing (10,000 iterations) incorporating spatial autocorrelation correction was performed, with FDR correction applied for multiple comparisons (*p_FDR_* < 0.05). Simultaneously, the Neurosynth platform was utilized to identify cognitive terms associated with the SC-FC coupling patterns. Using the platform's decoder tool, we computed correlation metrics (r) representing the association strength between the observed t-value maps and meta-analytically derived cognitive concepts. The top 10 most significantly associated cognitive terms were retained for subsequent analysis.

S9 Imaging-Transcriptomics Processing Pipeline
**Regional gene expression data were obtained from the** Allen Human Brain Atlas (AHBA)**. The abagen toolbox (https://github.com/netneurolab/abagen) was used to process AHBA data and map expression to 246 brain regions based on the BNA-246 atlas. Probe re-annotation and filtering followed established guidelines. Probes with intensity below background in >50% of samples were removed. Tissue samples were mapped to brain regions using MNI coordinates. A nearest-centroid approach was used for unmatched samples. Expression values were averaged per donor and region, then normalized using a robust sigmoid function. A final gene expression matrix was constructed with rows representing brain regions and columns** representing 15,633 genes. Only left-hemisphere cortical regions were included. PLS regression was used to relate regional gene expression to patterns of SC-FC coupling change in ALS-ES. The first PLS component (PLS1) captured maximum covariance between gene expression and structural changes. Explained variance was calculated for 1-15 components, with significance assessed via 1000 permutations. Bootstrap resampling (across 123 regions) estimated standard errors for gene weights; Z-scores were computed to rank gene contributions. Using a *p*_Bonferroni_ < 0.05 threshold, genes were classified as PLS1+ (Z > threshold) or PLS1− (Z < -threshold), indicating positive or negative associations with SC-FC coupling changes.

S10 Bioinformatics and Enrichment Analysis
We then used all genes in PLS1+ and PLS1- to conduct further bioinformatics analyses investigating whether these genes map to common and relevant biological pathways. First, Gene Ontology (GO) and Kyoto Encyclopedia of Genes and Genomes (KEGG) pathway annotations were performed using the clusterProfiler package. The GO terms and KEGG pathways with a corrected P-value < 0.05 were considered significantly enriched. After enrichment, all the significantly enriched GO terms were clustered based on semantic similarity by using the R package rrvgo (threshold = 0.7). Subsequently, the Metascape online platform (https://metascape.org) was employed for further pathway enrichment analysis of the significantly differentially expressed genes (*p*_FDR_ < 0.05). To identify cell-type enrichment of PLS1-weighted genes, we applied expression-weighted cell type enrichment (EWCE) analysis using two independent human single-cell transcriptomic datasets: the Allen Human Brain Atlas single-cell RNA sequencing (AHBA-seq) dataset and the DroNc-seq dataset.  Both datasets encompass major brain cell types, including astrocytes (ASC), microglia (MG), endothelial cells (ENDO), oligodendrocytes (ODC), oligodendrocyte precursor cells (OPC), and GABAergic neurons (GABA). Enrichment of PLS1+ and PLS1- gene sets in specific cell types was assessed and validated across both datasets. To associate PLS1-weighted genes with specific cortical layers, we performed multi-method enrichment analysis. Layer-specific gene expression profiles were derived from the Allen Human Brain Atlas, and single-sample gene set enrichment analysis (ssGSEA) was implemented via the Gene Set Variation Analysis (GSVA) package to quantify laminar enrichment scores using Gaussian regression. We further validated cortical layers specificity by intersecting PLS1-weighted genes with established cortical layers marker genes.

## S11 Single-Cell Analysis

To investigate cell-type-specific expression patterns in the motor cortex, we integrated and analyzed two publicly available single-cell RNA-seq datasets (GSE219281 and GSE174332). Data processing and analysis were performed using the Seurat R package. Rigorous quality control was applied to exclude low-quality cells: those expressing fewer than 200 genes, more than 10,000 genes, or with >20% mitochondrial gene content or >20% ribosomal gene content were filtered out. The remaining expression matrix was normalized using the LogNormalize method, and the top 2000 highly variable genes were selected using the FindVariableFeatures function. Gene expression values were scaled using the ScaleData function to achieve zero mean and unit variance. Linear dimensionality reduction was carried out via principal component analysis (PCA), followed by non-linear visualization using t-distributed Stochastic Neighbor Embedding (t-SNE) and Uniform Manifold Approximation and Projection (UMAP). Cell clusters were identified through an unsupervised approach using the FindNeighbors and FindClusters functions. Cell types were annotated automatically using the SingleR package by referencing curated transcriptomic datasets. We next extracted the gene sets most positively and negatively correlated with SC-FC coupling, as identified through PLS regression analysis. The expression patterns of these PLS-derived gene sets were systematically examined across different cell types in ALS and control motor cortex samples. GO and KEGG pathway enrichment analyses were then performed to interpret the biological significance of these expression signatures. Finally, we applied module scoring to evaluate the activity of these PLS-defined gene programs at single-cell resolution, enabling comparative assessment of their enrichment across neuronal and glial populations in disease and control conditions.

# Results

## S1 Structural Network Analysis

### S1.1 Global Topological Properties

Group comparisons showed no significant differences in global network metrics after FDR correction. Although uncorrected decreases in Cp (*p* = 0.021) and Eloc (*p* = 0.027) were observed in ALS-ES patients, these did not survive multiple comparisons adjustment (*p_FDR_* > 0.05). No other global topological measures reached significance (**Table S1**).

**Table S1. Global structural network topology comparisons between HCs and ALS-ES groups**

| **Variables** | **ALS-ES (n = 73)** | **HCs (n = 74)** | ***p*** | ***p*_FDR_** |
| --- | --- | --- | --- | --- |
| Cp | 0.356 ± 0.024 | 0.364 ± 0.025 | 0.021 | 0.137 |
| Lp | 3.176 ± 0.265 | 3.135 ± 0.196 | 0.282 | 0.672 |
| Eg | 0.317 ± 0.024 | 0.320 ± 0.019 | 0.336 | 0.672 |
| Eloc | 0.525 ± 0.051 | 0.540 ± 0.048 | 0.027 | 0.137 |
| Gamma | 8.349 ± 1.109 | 8.352 ± 0.989 | 0.921 | 0.976 |
| Lambda | 1.191 ± 0.031 | 1.194 ± 0.028 | 0.531 | 0.758 |
| Sigma | 6.993 ± 0.756 | 6.989 ± 0.729 | 0.976 | 0.976 |
| Assortativity | 0.162 ± 0.048 | 0.159 ± 0.049 | 0.501 | 0.758 |
| Hierarchy | 0.489 ± 0.062 | 0.486 ± 0.075 | 0.712 | 0.889 |
| Synchronization | 0.006 ± 0.007 | 0.008 ± 0.008 | 0.089 | 0.296 |

Abbreviations: HCs, healthy controls; ALS-ES, early stage amyotrophic lateral sclerosis; Cp, clustering coefficient; Lp, characteristic path length; Eg, global efficiency; Eloc, local efficiency; Gamma, normalized clustering coefficient; Lambda, normalized characteristic path length; Sigma, small-worldness; *p*_FDR_, false discovery rate corrected *p*-value.

### S1.2 Nodal Topological Properties

Regional analyses identified several brain regions showing Inter-group differences in nodal metrics including Bc, Dc, NCp, Ne, NLe, and NLp at the uncorrected significance level (*p* < 0.05). These regions were distributed across temporal, parietal, occipital, and frontal lobes, as well as subcortical structures. However, none of these regional differences survived FDR correction for multiple comparisons (*p_FDR_* > 0.05). The complete list of significant regional differences is provided in **Table S2**.

**Table S2. Nodal structural network topology comparisons between HCs and ALS-ES groups**

| **Variable** | **Brain region** | **Total (n = 147)** | **ALS-ES (n = 73)** | **HCs (n = 74)** | ***p*** |
| --- | --- | --- | --- | --- | --- |
| Bc | STG_R_6_6 | 187.769 ± 261.867 | 241.756 ± 307.809 | 134.511 ± 194.707 | 0.024 |
|  | ITG_R_7_2 | 33.193 ± 56.444 | 23.186 ± 40.652 | 43.064 ± 67.401 | 0.020 |
|  | ITG_R_7_4 | 158.916 ± 211.405 | 202.371 ± 274.228 | 116.048 ± 107.059 | 0.012 |
|  | PhG_L_6_4 | 14.697 ± 35.009 | 20.489 ± 45.692 | 8.982 ± 18.040 | 0.048 |
|  | SPL_L_5_1 | 534.002 ± 558.627 | 641.966 ± 640.840 | 427.497 ± 442.632 | 0.014 |
|  | CG_R_7_5 | 28.061 ± 46.380 | 21.335 ± 38.120 | 34.696 ± 52.717 | 0.037 |
|  | MVOcC _R_5_2 | 259.942 ± 354.681 | 179.835 ± 199.677 | 338.967 ± 446.682 | 0.013 |
|  | LOcC_R_4_4 | 247.969 ± 285.114 | 305.051 ± 330.921 | 191.658 ± 219.314 | 0.029 |
| Dc | OrG_R_6_4 | 9.626 ± 3.961 | 10.329 ± 4.246 | 8.932 ± 3.551 | 0.028 |
|  | PrG_L_6_2 | 8.952 ± 2.973 | 8.411 ± 2.833 | 9.486 ± 3.031 | 0.024 |
|  | PCL_L_2_1 | 6.633 ± 1.990 | 6.205 ± 2.020 | 7.054 ± 1.879 | 0.024 |
|  | STG_L_6_4 | 4.782 ± 2.462 | 4.397 ± 2.253 | 5.162 ± 2.612 | 0.037 |
|  | MTG_R_4_3 | 7.197 ± 3.557 | 6.644 ± 3.413 | 7.743 ± 3.634 | 0.039 |
|  | pSTS_R_2_1 | 6.667 ± 2.576 | 6.123 ± 2.236 | 7.203 ± 2.784 | 0.006 |
|  | SPL_R_5_4 | 9.973 ± 4.297 | 9.301 ± 3.925 | 10.635 ± 4.565 | 0.043 |
|  | PoG_R_4_2 | 6.408 ± 2.026 | 6.110 ± 1.671 | 6.703 ± 2.298 | 0.027 |
|  | INS_R_6_6 | 4.973 ± 3.016 | 4.438 ± 2.759 | 5.500 ± 3.181 | 0.017 |
|  | CG_R_7_3 | 2.796 ± 1.821 | 2.370 ± 1.663 | 3.216 ± 1.882 | 0.004 |
|  | MVOcC _R_5_2 | 8.633 ± 3.619 | 7.726 ± 2.621 | 9.527 ± 4.217 | 0.007 |
|  | MVOcC _R_5_5 | 10.190 ± 3.980 | 9.411 ± 3.265 | 10.959 ± 4.467 | 0.029 |
|  | LOcC _R_4_3 | 8.313 ± 3.925 | 7.767 ± 3.490 | 8.851 ± 4.267 | 0.034 |
|  | BG_L_6_6 | 19.707 ± 4.685 | 18.274 ± 4.171 | 21.122 ± 4.760 | 0.001 |
| NCp | STG_R_6_6 | 0.418 ± 0.195 | 0.374 ± 0.182 | 0.461 ± 0.199 | 0.008 |
|  | ITG_R_7_4 | 0.328 ± 0.145 | 0.300 ± 0.151 | 0.357 ± 0.135 | 0.039 |
|  | SPL_L_5_1 | 0.307 ± 0.128 | 0.286 ± 0.129 | 0.328 ± 0.124 | 0.039 |
|  | IPL_L_6_2 | 0.298 ± 0.090 | 0.284 ± 0.086 | 0.313 ± 0.093 | 0.049 |
|  | PoG_L_4_3 | 0.375 ± 0.144 | 0.351 ± 0.150 | 0.400 ± 0.135 | 0.045 |
|  | INS_R_6_1 | 0.431 ± 0.240 | 0.389 ± 0.230 | 0.472 ± 0.243 | 0.016 |
|  | CG_L_7_7 | 0.302 ± 0.109 | 0.286 ± 0.121 | 0.317 ± 0.093 | 0.044 |
|  | MVOcC _R_5_2 | 0.414 ± 0.144 | 0.446 ± 0.154 | 0.382 ± 0.126 | 0.007 |
|  | LOcC_R_4_4 | 0.360 ± 0.147 | 0.331 ± 0.110 | 0.389 ± 0.172 | 0.043 |
|  | LOcC _R_2_2 | 0.342 ± 0.135 | 0.318 ± 0.130 | 0.366 ± 0.136 | 0.038 |
| Ne | IFG_R_6_2 | 0.259 ± 0.066 | 0.249 ± 0.082 | 0.269 ± 0.041 | 0.044 |
|  | PrG_R_6_1 | 0.293 ± 0.031 | 0.287 ± 0.033 | 0.299 ± 0.029 | 0.041 |
|  | PrG_L_6_2 | 0.339 ± 0.036 | 0.332 ± 0.040 | 0.345 ± 0.030 | 0.037 |
|  | PrG_R_6_5 | 0.305 ± 0.030 | 0.299 ± 0.032 | 0.310 ± 0.027 | 0.030 |
|  | MTG_R_4_3 | 0.299 ± 0.038 | 0.294 ± 0.038 | 0.305 ± 0.037 | 0.047 |
|  | ITG_L_7_2 | 0.280 ± 0.032 | 0.275 ± 0.032 | 0.285 ± 0.032 | 0.037 |
|  | ITG_R_7_2 | 0.262 ± 0.042 | 0.254 ± 0.046 | 0.271 ± 0.035 | 0.015 |
|  | PoG_R_4_1 | 0.301 ± 0.030 | 0.296 ± 0.032 | 0.306 ± 0.027 | 0.039 |
|  | MVOcC _R_5_2 | 0.333 ± 0.040 | 0.325 ± 0.037 | 0.340 ± 0.041 | 0.048 |
| NLe | SFG_L_7_4 | 0.492 ± 0.150 | 0.464 ± 0.158 | 0.519 ± 0.137 | 0.021 |
|  | IFG_L_6_4 | 0.480 ± 0.342 | 0.537 ± 0.336 | 0.424 ± 0.341 | 0.031 |
|  | STG_L_6_4 | 0.532 ± 0.302 | 0.489 ± 0.319 | 0.575 ± 0.280 | 0.034 |
|  | STG_R_6_6 | 0.590 ± 0.220 | 0.551 ± 0.232 | 0.629 ± 0.201 | 0.038 |
|  | ITG_R_7_4 | 0.478 ± 0.210 | 0.433 ± 0.216 | 0.523 ± 0.195 | 0.017 |
|  | PCun_L_4_1 | 0.480 ± 0.197 | 0.444 ± 0.215 | 0.516 ± 0.172 | 0.044 |
|  | PoG_L_4_3 | 0.567 ± 0.176 | 0.535 ± 0.190 | 0.600 ± 0.156 | 0.024 |
|  | INS_R_6_1 | 0.563 ± 0.284 | 0.521 ± 0.285 | 0.604 ± 0.278 | 0.039 |
|  | LOcC_L_2_2 | 0.576 ± 0.189 | 0.546 ± 0.203 | 0.605 ± 0.171 | 0.032 |
|  | LOcC_R_2_2 | 0.543 ± 0.185 | 0.506 ± 0.191 | 0.580 ± 0.173 | 0.015 |
|  | Tha_R_8_3 | 0.620 ± 0.212 | 0.584 ± 0.242 | 0.656 ± 0.172 | 0.049 |
| NLp | IFG_R_6_2 | 2232.810 ± 11919.055 | 4492.330 ± 16667.070 | 3.823 ± 0.762 | 0.013 |
|  | PrG_R_6_1 | 3.459 ± 0.429 | 3.539 ± 0.491 | 3.379 ± 0.343 | 0.038 |
|  | PrG_R_6_5 | 3.314 ± 0.338 | 3.378 ± 0.372 | 3.250 ± 0.289 | 0.018 |
|  | PCL_L_2_1 | 3.156 ± 0.281 | 3.210 ± 0.320 | 3.101 ± 0.225 | 0.044 |
|  | MTG_R_4_3 | 3.398 ± 0.452 | 3.468 ± 0.488 | 3.328 ± 0.406 | 0.041 |
|  | ITG_L_7_2 | 3.620 ± 0.424 | 3.692 ± 0.438 | 3.549 ± 0.401 | 0.031 |
|  | PoG_R_4_1 | 3.359 ± 0.353 | 3.421 ± 0.391 | 3.297 ± 0.302 | 0.031 |
|  | MVOcC _L_5_5 | 2.844 ± 0.283 | 2.891 ± 0.327 | 2.798 ± 0.224 | 0.048 |
|  | Amyg_R_2_1 | 1786.719 ± 10698.249 | 3594.323 ± 15016.772 | 3.542 ± 0.351 | 0.033 |

Abbreviations: HCs, healthy controls; ALS-ES, early stage amyotrophic lateral sclerosis; Bc, betweenness centrality; Dc, degree centrality; NCp, nodal clustering coefficient; Ne, nodal efficiency; NLe, nodal local efficiency; NLp, nodal characteristic path length; L, left; R, right; STG, superior temporal gyrus; ITG, inferior temporal gyrus; PhG, parahippocampal gyrus; SPL, superior parietal lobule; CG, cingulate gyrus; MVOcC, medioventral occipital cortex; LOcC, lateral occipital cortex; OrG, orbital gyrus; PrG, precentral gyrus; PCL, paracentral lobule; MTG, middle temporal gyrus; pSTS, posterior superior temporal sulcus; PoG, postcentral gyrus; INS, insula; BG, basal ganglia; IFG, inferior frontal gyrus; SFG, superior frontal gyrus; PCun, precuneus; Tha, thalamus; Amyg, amygdala.

### S1.3 Intra-Network Modularity Analysis

No significant differences in intra-network structural connectivity were found between ALS-ES and HCs. All seven functional networks showed no statistically significant group differences. These results were consistent both before and after FDR correction with all *p_FDR_* values greater than 0.05 as detailed in Table S3.

**Table S3. Group comparisons of intra-network structural connectivity**

| **Variable** | **Total (n = 147)** | **ALS-ES (n = 73)** | **HCs (n = 74)** | ***p*** | ***p*_FDR_** |
| --- | --- | --- | --- | --- | --- |
| VIS | 0.393 ± 0.291 | 0.360 ± 0.142 | 0.426 ± 0.383 | 0.197 | 0.693 |
| SMN | 0.241 ± 0.109 | 0.237 ± 0.107 | 0.245 ± 0.113 | 0.732 | 0.732 |
| DAN | 0.213 ± 0.102 | 0.209 ± 0.097 | 0.217 ± 0.108 | 0.694 | 0.732 |
| VAN | 0.091 ± 0.080 | 0.083 ± 0.051 | 0.099 ± 0.101 | 0.275 | 0.693 |
| LIM | 0.162 ± 0.122 | 0.154 ± 0.077 | 0.170 ± 0.154 | 0.478 | 0.693 |
| FPN | 0.222 ± 0.120 | 0.213 ± 0.115 | 0.232 ± 0.125 | 0.405 | 0.693 |
| DMN | 0.091 ± 0.057 | 0.087 ± 0.045 | 0.095 ± 0.067 | 0.495 | 0.693 |

Abbreviations: HCs, healthy controls; ALS-ES, early stage amyotrophic lateral sclerosis; VIS, visual network; VAN, ventral attention network; FPN, frontoparietal network; LIM, limbic network; DMN, default mode network; DAN, dorsal attention network; SMN, somatomotor network; *p*_FDR_, false discovery rate corrected *p*-value.

### S1.4 Inter-Network Modularity Analysis

Analysis of inter-network structural connectivity revealed nominally significant decreases in the ALS-ES group compared to HCs for two network pairs: between the visual network and default mode network (VIS-DMN, *p*=0.040), and between the somatomotor network and default mode network (SMN-DMN, *p*=0.034). However, these differences did not survive FDR correction (*p_FDR_*>0.05). No other inter-network connections showed significant group differences. Complete results are provided in **Table S4**.

**Table S4. Group comparisons of inter-network structural connectivity**

| **Variable** | **Total (n = 147)** | **ALS-ES (n = 73)** | **HCs (n = 74)** | ***p*** | ***p*_FDR_** |
| --- | --- | --- | --- | --- | --- |
| VIS - SMN | 0.025 ± 0.016 | 0.025 ± 0.014 | 0.025 ± 0.017 | 0.950 | 0.950 |
| VIS - DAN | 0.104 ± 0.061 | 0.100 ± 0.052 | 0.107 ± 0.070 | 0.520 | 0.718 |
| VIS - VAN | 0.014 ± 0.015 | 0.013 ± 0.010 | 0.015 ± 0.018 | 0.356 | 0.718 |
| VIS - LIM | 0.089 ± 0.046 | 0.084 ± 0.037 | 0.095 ± 0.053 | 0.121 | 0.425 |
| VIS - FPN | 0.031 ± 0.026 | 0.032 ± 0.031 | 0.030 ± 0.022 | 0.547 | 0.718 |
| VIS - DMN | 0.063 ± 0.035 | 0.058 ± 0.029 | 0.069 ± 0.040 | 0.040* | 0.415 |
| SMN - DAN | 0.117 ± 0.058 | 0.109 ± 0.056 | 0.125 ± 0.059 | 0.064 | 0.425 |
| SMN - VAN | 0.093 ± 0.060 | 0.085 ± 0.050 | 0.100 ± 0.068 | 0.101 | 0.425 |
| SMN - LIM | 0.101 ± 0.068 | 0.093 ± 0.075 | 0.109 ± 0.061 | 0.186 | 0.555 |
| SMN - FPN | 0.052 ± 0.037 | 0.051 ± 0.038 | 0.053 ± 0.036 | 0.637 | 0.787 |
| SMN - DMN | 0.148 ± 0.076 | 0.134 ± 0.079 | 0.162 ± 0.071 | 0.034* | 0.415 |
| DAN - VAN | 0.054 ± 0.038 | 0.055 ± 0.039 | 0.054 ± 0.037 | 0.938 | 0.950 |
| DAN - LIM | 0.037 ± 0.025 | 0.035 ± 0.021 | 0.038 ± 0.028 | 0.538 | 0.718 |
| DAN - FPN | 0.158 ± 0.081 | 0.162 ± 0.080 | 0.154 ± 0.082 | 0.725 | 0.846 |
| DAN - DMN | 0.086 ± 0.052 | 0.086 ± 0.057 | 0.085 ± 0.046 | 0.817 | 0.903 |
| VAN - LIM | 0.028 ± 0.020 | 0.027 ± 0.020 | 0.029 ± 0.020 | 0.523 | 0.718 |
| VAN - FPN | 0.077 ± 0.039 | 0.075 ± 0.043 | 0.079 ± 0.035 | 0.409 | 0.718 |
| VAN - DMN | 0.178 ± 0.103 | 0.162 ± 0.093 | 0.193 ± 0.110 | 0.107 | 0.425 |
| LIM - FPN | 0.063 ± 0.040 | 0.067 ± 0.040 | 0.058 ± 0.041 | 0.256 | 0.597 |
| LIM - DMN | 0.060 ± 0.036 | 0.056 ± 0.027 | 0.063 ± 0.044 | 0.212 | 0.555 |
| FPN - DMN | 0.101 ± 0.057 | 0.098 ± 0.057 | 0.103 ± 0.058 | 0.512 | 0.718 |

Abbreviations: HCs, healthy controls; ALS-ES, early stage amyotrophic lateral sclerosis; VIS, visual network; SMN, somatomotor network; DAN, dorsal attention network; VAN, ventral attention network; LIM, limbic network; FPN, frontoparietal network; DMN, default mode network; *p*_FDR_, false discovery rate corrected *p*-value.

## S2 Functional Network Analysis

### S2.1 Global Topological Properties

Group comparisons showed no significant differences in global functional network metrics after FDR correction. Although an uncorrected increase in Synchronization (*p* = 0.012) was observed in ALS-ES patients, it did not survive multiple comparisons adjustment (*p_FDR_* = 0.119). No other global topological measures reached statistical significance (**Table S5**).

**Table S5. Global functional network topology comparisons between HCs and ALS-ES groups**

| **Variables** | **ALS-ES (n = 73)** | **HCs (n = 74)** | ***p*** | ***p*_FDR_** |
| --- | --- | --- | --- | --- |
| Cp | 0.275 ± 0.013 | 0.277 ± 0.012 | 0.166 | 0.331 |
| Lp | 0.810 ± 0.043 | 0.811 ± 0.036 | 0.850 | 0.948 |
| Eg | 0.265 ± 0.008 | 0.265 ± 0.006 | 0.913 | 0.948 |
| Eloc | 0.354 ± 0.005 | 0.355 ± 0.005 | 0.133 | 0.331 |
| Gamma | 0.773 ± 0.123 | 0.738 ± 0.104 | 0.086 | 0.331 |
| Lambda | 0.482 ± 0.012 | 0.482 ± 0.011 | 0.948 | 0.948 |
| Sigma | 0.707 ± 0.118 | 0.677 ± 0.101 | 0.126 | 0.331 |
| Assortativity | 0.112 ± 0.037 | 0.112 ± 0.034 | 0.936 | 0.948 |
| Hierarchy | 0.019 ± 0.032 | 0.017 ± 0.028 | 0.740 | 0.948 |
| Synchronization | 0.013 ± 0.011 | 0.009 ± 0.009 | 0.012* | 0.119 |

Abbreviations: HCs, healthy controls; ALS-ES, early stage amyotrophic lateral sclerosis; Cp, clustering coefficient; Lp, characteristic path length; Eg, global efficiency; Eloc, local efficiency; Gamma, normalized clustering coefficient; Lambda, normalized characteristic path length; Sigma, small-worldness; *p*_FDR_, false discovery rate corrected *p*-value.

### S2.2 Nodal Topological Properties

Analysis of nodal functional network topology revealed significant between-group differences in several brain regions. Specifically, the ALS-ES group showed altered Bc in the right superior parietal lobule (SPL) and right postcentral gyrus (PoG), decreased Dc and Ne along with increased NLp in the left SPL, and decreased Ne with increased NLp in the left PoG. All these differences survived FDR correction (*p_FDR_* < 0.05). Complete results are provided in **Table S6**.

**Table S6. Nodal functional network topology comparisons between HCs and ALS-ES groups**

| **Variable** | **Brain region** | **Total (n = 147)** | **ALS-ES (n = 73)** | **HCs (n = 74)** | ***p*** | ***p*_FDR_** |
| --- | --- | --- | --- | --- | --- | --- |
| aBc | SPL_R_5_5 | 45.357 ± 32.890 | 54.924 ± 34.773 | 35.919 ± 28.090 | *p*<0.001 | 0.035 |
|  | PoG_R_4_3 | 38.635 ± 31.176 | 47.275 ± 35.412 | 30.112 ± 23.638 | *p*<0.001 | 0.035 |
| aDc | SPL_L_5_4 | 40.277 ± 8.990 | 37.184 ± 8.306 | 43.328 ± 8.638 | *p*<0.001 | 0.015 |
| aNe | SPL_L_5_4 | 0.293 ± 0.020 | 0.286 ± 0.018 | 0.300 ± 0.020 | *p*<0.001 | 0.006 |
|  | PoG_L_4_4 | 0.310 ± 0.021 | 0.304 ± 0.021 | 0.316 ± 0.018 | *p*<0.001 | 0.029 |
| aNLp | SPL_L_5_4 | 0.720 ± 0.054 | 0.739 ± 0.052 | 0.701 ± 0.050 | *p*<0.001 | 0.005 |
|  | PoG_L_4_1 | 0.767 ± 0.092 | 0.791 ± 0.103 | 0.742 ± 0.072 | *p*<0.001 | 0.033 |
|  | PoG_L_4_4 | 0.681 ± 0.051 | 0.697 ± 0.055 | 0.665 ± 0.041 | *p*<0.001 | 0.008 |

Abbreviations: HCs, healthy controls; ALS-ES, early stage amyotrophic lateral sclerosis; Bc, betweenness centrality; Dc, degree centrality; Ne, nodal efficiency; NLp, nodal characteristic path length; L, left; R, right; *p*_FDR_, false discovery rate corrected *p*-value.

### S2.3 Intra-Network Modularity Analysis

Analysis of Intra-network functional connectivity revealed a statistically significant decrease in the SMN in ALS-ES patients compared to HCs (*p_FDR_* = 0.008). A nominally significant reduction was also observed in the VIS(*p* = 0.049), though this did not survive FDR correction (*p_FDR_* = 0.064). No other functional networks showed significant inter-group differences. Complete results are presented in **Table S7**.

**Table S7. Group comparisons of intra-network functional connectivity**

| **Brain region** | **Total (n = 147)** | **ALS-ES (n = 73)** | **HCs (n = 74)** | ***p*** | ***p*_FDR_** |
| --- | --- | --- | --- | --- | --- |
| VIS | 147.295 ± 29.867 | 142.706 ± 28.254 | 151.821 ± 30.902 | 0.049 | 0.064 |
| SMN | 165.043 ± 31.56 | 158.149 ± 28.871 | 171.845 ± 32.794 | 0.006 | 0.008 |
| DAN | 124.715 ± 17.684 | 124.354 ± 18.556 | 125.071 ± 16.899 | 0.757 | 0.807 |
| VAN | 55.18 ± 11.356 | 54.328 ± 10.833 | 56.02 ± 11.864 | 0.184 | 0.368 |
| LIM | 59.1 ± 18.64 | 57.961 ± 19.373 | 60.225 ± 17.949 | 0.441 | 0.464 |
| FPN | 62.143 ± 13.413 | 63.666 ± 13.708 | 60.641 ± 13.034 | 0.273 | 0.172 |
| DMN | 128.85 ± 29.082 | 126.608 ± 29.19 | 131.061 ± 29.003 | 0.280 | 0.355 |

Abbreviations: HCs, healthy controls; ALS-ES, early stage amyotrophic lateral sclerosis; VIS, visual network; VAN, ventral attention network; FPN, frontoparietal network; LIM, limbic network; DMN, default mode network; DAN, dorsal attention network; SMN, somatomotor network; *p*_FDR_, false discovery rate corrected *p*-value.

### S2.4 Inter-Network Modularity Analysis

Analysis of inter-network functional connectivity revealed several nominally significant alterations in the ALS-ES group. Specifically, decreased connectivity was observed in the following network pairs: between the visual network and somatomotor network (VIS-SMN, *p* = 0.031), between the visual network and frontoparietal network (VIS-FPN, *p* = 0.031), between the visual network and default mode network (VIS-DMN, *p* = 0.003), between the dorsal attention network and frontoparietal network (DAN-FPN, *p* = 0.017), and between the dorsal attention network and default mode network (DAN-DMN, *p* = 0.022). However, none of these differences survived FDR correction (all *p_FDR_* > 0.05). No other inter-network connections showed statistically significant group differences. Complete results are provided in **Table S8**.

**Table S8. Group comparisons of inter-network functional connectivity**

| **Variable** | **Total (n = 147)** | **ALS-ES (n = 73)** | **HCs (n = 74)** | **p** | ***p*_FDR_** |
| --- | --- | --- | --- | --- | --- |
| VIS - SMN | 181.733 ± 69.233 | 168.786 ± 65.372 | 194.506 ± 70.982 | 0.031* | 0.131 |
| VIS - DAN | 177.958 ± 56.443 | 172.827 ± 50.843 | 183.02 ± 61.398 | 0.258 | 0.503 |
| VIS - VAN | 62.406 ± 24.277 | 63.124 ± 24.446 | 61.697 ± 24.255 | 0.624 | 0.765 |
| VIS - LIM | 76.692 ± 32.344 | 75.673 ± 29.288 | 77.697 ± 35.272 | 0.899 | 0.899 |
| VIS - FPN | 79.282 ± 24.131 | 83.322 ± 23.702 | 75.297 ± 24.045 | 0.031* | 0.131 |
| VIS - DMN | 123.39 ± 41.598 | 132.124 ± 40.451 | 114.774 ± 41.176 | 0.003* | 0.056 |
| SMN - DAN | 217.137 ± 54.007 | 210.453 ± 51.645 | 223.73 ± 55.805 | 0.184 | 0.484 |
| SMN - VAN | 151.703 ± 37.126 | 150.297 ± 34.248 | 153.091 ± 39.949 | 0.499 | 0.699 |
| SMN - LIM | 72.027 ± 35.361 | 66.749 ± 33.137 | 77.234 ± 36.911 | 0.099 | 0.346 |
| SMN - FPN | 82.052 ± 27.955 | 80.758 ± 25.337 | 83.328 ± 30.438 | 0.683 | 0.765 |
| SMN - DMN | 105.072 ± 45.703 | 101.485 ± 41.887 | 108.611 ± 49.209 | 0.407 | 0.657 |
| DAN - VAN | 95.371 ± 29.345 | 98.714 ± 30.864 | 92.073 ± 27.578 | 0.229 | 0.503 |
| DAN - LIM | 73.274 ± 29.241 | 72.854 ± 27.849 | 73.687 ± 30.737 | 0.842 | 0.885 |
| DAN - FPN | 126.813 ± 26.258 | 131.628 ± 26.619 | 122.063 ± 25.18 | 0.017* | 0.131 |
| DAN - DMN | 100.434 ± 29.422 | 104.768 ± 32.331 | 96.159 ± 25.749 | 0.022* | 0.131 |
| VAN - LIM | 31.519 ± 13.695 | 30.613 ± 14.096 | 32.414 ± 13.322 | 0.440 | 0.661 |
| VAN - FPN | 62.94 ± 16.82 | 63.7 ± 18.104 | 62.191 ± 15.537 | 0.692 | 0.765 |
| VAN - DMN | 67.895 ± 19.593 | 65.427 ± 18.352 | 70.329 ± 20.579 | 0.130 | 0.390 |
| LIM - FPN | 58.765 ± 21.413 | 60.324 ± 21.676 | 57.227 ± 21.185 | 0.326 | 0.570 |
| LIM - DMN | 101.28 ± 32.01 | 99.456 ± 32.658 | 103.079 ± 31.477 | 0.634 | 0.765 |
| FPN - DMN | 131.464 ± 30.403 | 134.868 ± 31.322 | 128.106 ± 29.291 | 0.263 | 0.503 |

Abbreviations: HCs, healthy controls; ALS-ES, early stage amyotrophic lateral sclerosis; VIS, visual network; VAN, ventral attention network; FPN, frontoparietal network; LIM, limbic network; DMN, default mode network; DAN, dorsal attention network; SMN, somatomotor network; *p*_FDR_, false discovery rate corrected *p*-value.

## S3 SC-FC coupling Analysis

### S3.1 Global and Regional SC-FC Coupling

Global-level analysis revealed SC-FC coupling values of 0.081 in HCs and 0.073 in ALS-ES patients, indicating a non-significant reduction in the ALS-ES group (*p* = 0.37). At the regional level, significant between-group differences in SC-FC coupling were identified after FDR correction (*p* < 0.05). Specifically, ALS-ES patients exhibited significantly decreased coupling in regions including the SFG_L_7_6 (ALS: 0.169 ± 0.061 vs. HCs: 0.204 ± 0.052, *p*_FDR_=0.009), PrG_L_6_1 (0.115 ± 0.053 vs. 0.170 ± 0.043, *p*_FDR_<0.001), and CG_R_7_5 (0.070 ± 0.067 vs. 0.116 ± 0.062, *p*_FDR_=0.009), among others (**Table S9**).

**Table S9. Comparison of SC-FC coupling values at the brain region level**

| **Brain region** | **Total (n = 147)** | **ALS-ES (n = 73)** | **HCs (n = 74)** | ***p*** | ***p*_FDR_** |
| --- | --- | --- | --- | --- | --- |
| SFG_L_7_6 | 0.186 ± 0.059 | 0.169 ± 0.061 | 0.204 ± 0.052 | p<0.001 | 0.009 |
| IFG_R_6_2 | 0.119 ± 0.057 | 0.103 ± 0.054 | 0.134 ± 0.055 | 0.001 | 0.045 |
| IFG_L_6_6 | 0.119 ± 0.067 | 0.102 ± 0.067 | 0.136 ± 0.063 | 0.001 | 0.045 |
| PrG_L_6_1 | 0.142 ± 0.056 | 0.115 ± 0.053 | 0.170 ± 0.043 | p<0.001 | p<0.001 |
| CG_R_7_3 | 0.120 ± 0.062 | 0.103 ± 0.058 | 0.136 ± 0.063 | 0.002 | 0.045 |
| CG_R_7_5 | 0.094 ± 0.069 | 0.070 ± 0.067 | 0.116 ± 0.062 | p<0.001 | 0.009 |
| LOcC_L_4_3 | 0.178 ± 0.064 | 0.162 ± 0.060 | 0.195 ± 0.063 | 0.002 | 0.045 |
| BG_R_6_5 | 0.157 ± 0.085 | 0.125 ± 0.070 | 0.129 ± 0.090 | p<0.001 | 0.009 |
| Tha_L_8_3 | 0.110 ± 0.069 | 0.092 ± 0.068 | 0.127 ± 0.067 | 0.001 | 0.045 |

Abbreviations: HCs, healthy controls; ALS-ES, early stage amyotrophic lateral sclerosis; SFG, Superior Frontal Gyrus; IFG, Inferior Frontal Gyrus; PrG, Precentral Gyrus; CG, Cingulate Gyrus; LOcC, Lateral Occipital Cortex; BG, Basal Ganglia; Tha, Thalamus; L, Left; R, Right; *p*_FDR_, false discovery rate corrected *p*-value.

### S3.2 Intra- and Inter-Network SC-FC Coupling

Analysis of intra-network SC-FC coupling, adjusted for age, sex, and TIV, revealed a significant reduction in the SMN of ALS-ES patients (0.279 ± 0.037) compared to HCs (0.302 ± 0.041; *p*_FDR_ = 0.001). Nominally significant uncorrected differences were also observed in the DMN (*p* = 0.017) and VIS (*p* = 0.024), though these did not reach statistical significance after FDR correction (**Table S10**). Analysis of inter-network coupling revealed more selective alterations after FDR correction. Specifically, ALS-ES patients showed significantly reduced coupling between the SMN and the VAN (*p*_FDR_ = 0.032) and LIM (*p*_FDR_ = 0.032), as well as between the VAN and the FPN (*p*_FDR_ = 0.032) and DMN (*p*_FDR_ = 0.032). Nominally significant uncorrected differences (*p* < 0.05) were observed for the connections between the SMN and DAN (*p* = 0.017) and between the DAN and VAN (*p* = 0.022), although these did not survive multiple comparisons correction (**Table S11**). Effect size analysis revealed that the magnitude of SC-FC decoupling was highest in the SMN (Cohen’s *d* = 0.605), nearly doubling the average effect size seen across other functional networks (mean *d* = 0.230), further supporting the regional vulnerability of the motor system (**Fig. S1a)**. The robustness of the SMN decoupling was further validated using a permutation test (5,000 iterations), confirming that the observed reduction was significantly greater than chance (T = 2.833, *p*_perm_ = 0.004) (**Fig. S1b)**.

**Fig. S1 Robustness and effect size of SC-FC decoupling.** (a) Somatomotor network shows the highest effect size among canonical networks, indicating its primary vulnerability. (b) Permutation testing confirms that the observed network decoupling is significantly greater than would be expected by chance.

**Table S10. Comparison of SC-FC coupling values at the intra-network**

| **Network** | **Total (n = 147)** | **ALS-ES (n = 73)** | **HCs (n = 74)** | ***p*** | ***p*_FDR_** |
| --- | --- | --- | --- | --- | --- |
| VIS | 0.313 ± 0.050 | 0.304 ± 0.056 | 0.322 ± 0.043 | 0.024* | 0.057 |
| SMN | 0.291 ± 0.041 | 0.279 ± 0.037 | 0.302 ± 0.041 | 0.000* | 0.001* |
| DAN | 0.231 ± 0.044 | 0.226 ± 0.046 | 0.236 ± 0.043 | 0.183 | 0.256 |
| VAN | 0.215 ± 0.057 | 0.212 ± 0.058 | 0.219 ± 0.056 | 0.535 | 0.624 |
| LIM | 0.260 ± 0.050 | 0.257 ± 0.054 | 0.262 ± 0.047 | 0.664 | 0.664 |
| FPN | 0.230 ± 0.055 | 0.225 ± 0.057 | 0.236 ± 0.052 | 0.169 | 0.256 |
| DMN | 0.199 ± 0.042 | 0.192 ± 0.045 | 0.207 ± 0.037 | 0.017* | 0.057 |

Abbreviations: HCs, healthy controls; ALS-ES, early stage amyotrophic lateral sclerosis; VIS, visual network; VAN, ventral attention network; FPN, frontoparietal network; LIM, limbic network; DMN, default mode network; DAN, dorsal attention network; SMN, somatomotor network; *p*_FDR_, false discovery rate corrected *p*-value.

**Table S11. Comparison of SC-FC coupling values at the inter-network**

| **Network** | **Total (n = 147)** | **ALS-ES (n = 73)** | **HCs (n = 74)** | ***p*** | ***p*_FDR_** |
| --- | --- | --- | --- | --- | --- |
| VIS - SMN | 0.075 ± 0.036 | 0.072 ± 0.038 | 0.077 ± 0.033 | 0.528 | 0.792 |
| VIS - DAN | 0.150 ± 0.037 | 0.149 ± 0.035 | 0.151 ± 0.040 | 0.646 | 0.843 |
| VIS - VAN | 0.066 ± 0.045 | 0.064 ± 0.045 | 0.068 ± 0.045 | 0.638 | 0.843 |
| VIS - LIM | 0.104 ± 0.043 | 0.107 ± 0.042 | 0.101 ± 0.043 | 0.457 | 0.738 |
| VIS - FPN | 0.086 ± 0.037 | 0.080 ± 0.034 | 0.091 ± 0.039 | 0.113 | 0.264 |
| VIS - DMN | 0.136 ± 0.036 | 0.134 ± 0.039 | 0.138 ± 0.033 | 0.786 | 0.843 |
| SMN - DAN | 0.157 ± 0.038 | 0.149 ± 0.038 | 0.165 ± 0.036 | 0.017 | 0.072 |
| SMN - VAN | 0.181 ± 0.039 | 0.172 ± 0.036 | 0.190 ± 0.040 | 0.005 | 0.032* |
| SMN - LIM | 0.146 ± 0.033 | 0.138 ± 0.030 | 0.153 ± 0.033 | 0.003 | 0.032* |
| SMN - FPN | 0.063 ± 0.039 | 0.059 ± 0.038 | 0.068 ± 0.040 | 0.247 | 0.432 |
| SMN - DMN | 0.148 ± 0.029 | 0.145 ± 0.026 | 0.151 ± 0.031 | 0.238 | 0.432 |
| DAN - VAN | 0.120 ± 0.040 | 0.115 ± 0.041 | 0.126 ± 0.038 | 0.022 | 0.076 |
| DAN - LIM | 0.075 ± 0.041 | 0.077 ± 0.041 | 0.073 ± 0.040 | 0.704 | 0.843 |
| DAN - FPN | 0.175 ± 0.040 | 0.168 ± 0.047 | 0.181 ± 0.032 | 0.075 | 0.224 |
| DAN - DMN | 0.107 ± 0.038 | 0.103 ± 0.037 | 0.112 ± 0.039 | 0.087 | 0.228 |
| VAN - LIM | 0.091 ± 0.039 | 0.090 ± 0.041 | 0.092 ± 0.038 | 0.753 | 0.843 |
| VAN - FPN | 0.102 ± 0.048 | 0.090 ± 0.047 | 0.115 ± 0.045 | 0.003 | 0.032* |
| VAN - DMN | 0.186 ± 0.040 | 0.176 ± 0.035 | 0.195 ± 0.043 | 0.006 | 0.032* |
| LIM - FPN | 0.16 ± 0.041 | 0.154 ± 0.045 | 0.166 ± 0.037 | 0.145 | 0.304 |
| LIM - DMN | 0.158 ± 0.041 | 0.157 ± 0.041 | 0.158 ± 0.041 | 0.880 | 0.880 |
| FPN - DMN | 0.168 ± 0.037 | 0.168 ± 0.040 | 0.168 ± 0.035 | 0.803 | 0.843 |

Abbreviations: HCs, healthy controls; ALS-ES, early stage amyotrophic lateral sclerosis; VIS, visual network; VAN, ventral attention network; FPN, frontoparietal network; LIM, limbic network; DMN, default mode network; DAN, dorsal attention network; SMN, somatomotor network; *p*_FDR_, false discovery rate corrected *p*-value.

### S3.3 Sensitivity Analysis of Structural-Functional Coupling

To further validate the robustness of our findings, we conducted a comprehensive sensitivity analysis by incorporating a full set of potential confounders into the general linear models. Specifically, age, sex, total intracranial volume (TIV), mean head motion (FD), and affective symptoms (HARS and HDRS scores) were simultaneously controlled as covariates. As detailed in **Tables S12-S14**, the significant SC-FC coupling alterations remained highly consistent with our primary results. At the regional level, the left precentral gyrus (*p_FDR_* < 0.001) and cingulate gyrus continued to show significant decoupling. At the network level, intra-network decoupling within the somatomotor network (SMN, *p_FDR_* = 0.024) and inter-network decoupling involving the SMN remained statistically significant. These supplementary results confirm that the observed network dysfunction is a robust neurobiological signature of early-stage ALS and is independent of individual variations in head motion or emotional states.

**Table S12. Comparison of SC-FC coupling values at the brain region level**

| **Brain region** | **Total (n = 147)** | **ALS-ES (n = 73)** | **HCs (n = 74)** | ***p*** | ***p*_FDR_** |
| --- | --- | --- | --- | --- | --- |
| PrG_L_6_1 | 0.142 ± 0.056 | 0.115 ± 0.053 | 0.170 ± 0.043 | ＜0.001 | ＜0.001 |
| LOcC_L_4_3 | 0.178 ± 0.064 | 0.162 ± 0.060 | 0.195 ± 0.063 | ＜0.001 | 0.001 |
| CG_R_7_5 | 0.094 ± 0.069 | 0.070 ± 0.067 | 0.116 ± 0.062 | ＜0.001 | 0.035 |
| CG_L_7_3 | 0.160 ± 0.055 | 0.146 ± 0.059 | 0.174 ± 0.048 | 0.001 | 0.047 |
| CG_R_7_3 | 0.120 ± 0.062 | 0.103 ± 0.058 | 0.136 ± 0.063 | 0.001 | 0.047 |
| PrG_L_6_6 | 0.167 ± 0.063 | 0.151 ± 0.066 | 0.182 ± 0.056 | 0.001 | 0.048 |
| BG_R_6_5 | 0.157 ± 0.085 | 0.185 ± 0.070 | 0.129 ± 0.090 | 0.001 | 0.048 |

Abbreviations: HCs, healthy controls; ALS-ES, early stage amyotrophic lateral sclerosis; PrG, Precentral Gyrus; LOcC, Lateral Occipital Cortex; CG, Cingulate Gyrus; BG, Basal Ganglia; L, Left; R, Right; *p*_FDR_, false discovery rate corrected *p*-value.

**Table S13. Comparison of SC-FC coupling values at the intra-network**

| **Network** | **Total (n = 147)** | **ALS-ES (n = 73)** | **HCs (n = 74)** | ***p*** | ***p*_FDR_** |
| --- | --- | --- | --- | --- | --- |
| VIS | 0.313 ± 0.050 | 0.304 ± 0.056 | 0.322 ± 0.043 | 0.143 | 0.200 |
| SMN | 0.291 ± 0.041 | 0.279 ± 0.037 | 0.302 ± 0.041 | 0.003* | 0.024* |
| DAN | 0.231 ± 0.044 | 0.226 ± 0.046 | 0.236 ± 0.043 | 0.023* | 0.082 |
| VAN | 0.215 ± 0.057 | 0.212 ± 0.058 | 0.219 ± 0.056 | 0.589 | 0.594 |
| LIM | 0.260 ± 0.050 | 0.257 ± 0.054 | 0.262 ± 0.047 | 0.594 | 0.594 |
| FPN | 0.230 ± 0.055 | 0.225 ± 0.057 | 0.236 ± 0.052 | 0.129 | 0.200 |
| DMN | 0.199 ± 0.042 | 0.192 ± 0.045 | 0.207 ± 0.037 | 0.089 | 0.200 |

Abbreviations: HCs, healthy controls; ALS-ES, early stage amyotrophic lateral sclerosis; VIS, visual network; VAN, ventral attention network; FPN, frontoparietal network; LIM, limbic network; DMN, default mode network; DAN, dorsal attention network; SMN, somatomotor network; *p*_FDR_, false discovery rate corrected *p*-value.

**Table S14. Comparison of SC-FC coupling values at the inter-network**

| **Network** | **Total (n = 147)** | **ALS-ES (n = 73)** | **HCs (n = 74)** | ***p*** | ***p*_FDR_** |
| --- | --- | --- | --- | --- | --- |
| VIS - SMN | 0.075 ± 0.036 | 0.072 ± 0.038 | 0.077 ± 0.033 | 0.949 | 0.949 |
| VIS - DAN | 0.150 ± 0.037 | 0.149 ± 0.035 | 0.151 ± 0.040 | 0.863 | 0.907 |
| VIS - VAN | 0.066 ± 0.045 | 0.064 ± 0.045 | 0.068 ± 0.045 | 0.387 | 0.646 |
| VIS - LIM | 0.104 ± 0.043 | 0.107 ± 0.042 | 0.101 ± 0.043 | 0.584 | 0.681 |
| VIS - FPN | 0.086 ± 0.037 | 0.080 ± 0.034 | 0.091 ± 0.039 | 0.034 | 0.102 |
| VIS - DMN | 0.136 ± 0.036 | 0.134 ± 0.039 | 0.138 ± 0.033 | 0.376 | 0.646 |
| SMN - DAN | 0.157 ± 0.038 | 0.149 ± 0.038 | 0.165 ± 0.036 | 0.009 | 0.036* |
| SMN - VAN | 0.181 ± 0.039 | 0.172 ± 0.036 | 0.190 ± 0.040 | 0.007 | 0.036* |
| SMN - LIM | 0.146 ± 0.033 | 0.138 ± 0.030 | 0.153 ± 0.033 | 0.009 | 0.036* |
| SMN - FPN | 0.063 ± 0.039 | 0.059 ± 0.038 | 0.068 ± 0.040 | 0.457 | 0.678 |
| SMN - DMN | 0.148 ± 0.029 | 0.145 ± 0.026 | 0.151 ± 0.031 | 0.484 | 0.678 |
| DAN - VAN | 0.120 ± 0.040 | 0.115 ± 0.041 | 0.126 ± 0.038 | 0.094 | 0.219 |
| DAN - LIM | 0.075 ± 0.041 | 0.077 ± 0.041 | 0.073 ± 0.040 | 0.356 | 0.646 |
| DAN - FPN | 0.175 ± 0.040 | 0.168 ± 0.047 | 0.181 ± 0.032 | 0.065 | 0.171 |
| DAN - DMN | 0.107 ± 0.038 | 0.103 ± 0.037 | 0.112 ± 0.039 | 0.032 | 0.102 |
| VAN - LIM | 0.091 ± 0.039 | 0.090 ± 0.041 | 0.092 ± 0.038 | 0.557 | 0.681 |
| VAN - FPN | 0.102 ± 0.048 | 0.090 ± 0.047 | 0.115 ± 0.045 | 0.001 | 0.024* |
| VAN - DMN | 0.186 ± 0.040 | 0.176 ± 0.035 | 0.195 ± 0.043 | 0.007 | 0.036* |
| LIM - FPN | 0.16 ± 0.041 | 0.154 ± 0.045 | 0.166 ± 0.037 | 0.570 | 0.681 |
| LIM - DMN | 0.158 ± 0.041 | 0.157 ± 0.041 | 0.158 ± 0.041 | 0.764 | 0.844 |
| FPN - DMN | 0.168 ± 0.037 | 0.168 ± 0.040 | 0.168 ± 0.035 | 0.400 | 0.646 |

Abbreviations: HCs, healthy controls; ALS-ES, early stage amyotrophic lateral sclerosis; VIS, visual network; VAN, ventral attention network; FPN, frontoparietal network; LIM, limbic network; DMN, default mode network; DAN, dorsal attention network; SMN, somatomotor network; *p*_FDR_, false discovery rate corrected *p*-value.

## S4 Machine Learning Analysis

### S4.1 Feature Selection

A total of 3,302 features were initially extracted, including: global topological properties of the DTI network (n=10); local topological properties of 246 brain regions in the DTI network (246×6=1,476); intra-network connectivity of the DTI network (n=7); inter-network connectivity of the DTI network (n=21); global topological properties of the fMRI network (n=10); local topological properties of 246 brain regions in the fMRI network (246×6=1,476); intra-network connectivity of the fMRI network (n=7); inter-network connectivity of the fMRI network (n=21); structural-functional connectivity coupling values of brain regions (n=246); intra-network structural-functional connectivity coupling values (n=7); and inter-network structural-functional connectivity coupling values (n=21). After removing missing values, 3,036 features remained. The dataset was divided into training and testing sets at a 7:3 ratio. A two-stage feature selection strategy was implemented: first, t-tests identified 76 significant features, which were subsequently incorporated into LASSO regression. This final selection yielded 29 optimal features. The values of these 29 features in both training and testing sets are presented in **Table S15**, showing no significant differences between the two sets.

**Table S15. Characteristics and LASSO coefficients of the selected features**

| **Variable** | **Train (Mean ± SD)** | **Test (Mean ± SD)** | **LASSO Coefficient** | ***p*** |
| --- | --- | --- | --- | --- |
| DTI_BC_STG_R | 198.88 (269.43) | 159.99 (242.79) | 0.000 | 0.418 |
| DTI_BC_SPL_R | 172.39 (198.04) | 199.57 (214.01) | 0.000 | 0.464 |
| DTI_DC_PCL_L | 6.79 (2.04) | 6.24 (1.83) | -0.016 | 0.129 |
| DTI_DC_pSTS_R | 6.52 (2.52) | 7.02 (2.72) | -0.015 | 0.289 |
| DTI_DC_BG_L | 19.61 (4.68) | 19.95 (4.76) | -0.002 | 0.690 |
| DTI_NCP_SPL_R | 0.40 (0.16) | 0.39 (0.15) | -0.252 | 0.667 |
| DTI_NCP_IPL_L | 0.30 (0.08) | 0.30 (0.11) | -0.187 | 0.841 |
| DTI_NCP_INS_R | 0.39 (0.34) | 0.49 (0.35) | -0.073 | 0.091 |
| DTI_NCP_LOcC_R | 0.37 (0.19) | 0.38 (0.18) | -0.226 | 0.860 |
| DTI_NLE_IFG_L | 0.52 (0.33) | 0.38 (0.35) | 0.092 | 0.029 |
| fMRI_BC_MFG_L | 41.22 (28.85) | 35.73 (27.40) | 0.000 | 0.292 |
| fMRI_BC_SPL_R | 42.63 (32.49) | 52.18 (33.29) | 0.000 | 0.112 |
| fMRI_BC_PoG_R | 37.87 (30.75) | 40.56 (32.52) | 0.001 | 0.638 |
| fMRI_BC_INS_R | 23.80 (26.06) | 23.91 (22.37) | 0.001 | 0.980 |
| fMRI_BC_CG_L | 83.68 (57.85) | 87.31 (59.41) | 0.000 | 0.734 |
| fMRI_BC_BG_R | 33.76 (44.37) | 31.42 (37.58) | 0.001 | 0.763 |
| fMRI_DC_SFG_L | 25.14 (9.59) | 25.07 (10.45) | 0.001 | 0.967 |
| fMRI_DC_MFG_R | 31.65 (11.31) | 28.20 (12.73) | 0.003 | 0.109 |
| fMRI_DC_IPL_R | 28.20 (10.77) | 27.14 (9.12) | 0.002 | 0.575 |
| fMRI_NCP_pSTS_R | 0.29 (0.04) | 0.29 (0.04) | -0.953 | 0.589 |
| fMRI_NCP_LOcC_L | 0.33 (0.05) | 0.34 (0.05) | -0.189 | 0.499 |
| fMRI_NE_PoG_L | 0.28 (0.03) | 0.28 (0.03) | -0.991 | 0.310 |
| fMRI_NE_PoG_R | 0.29 (0.03) | 0.28 (0.03) | -0.228 | 0.084 |
| fMRI_NE_PoG_L | 0.31 (0.02) | 0.31 (0.02) | -2.689 | 0.305 |
| fMRI_NE_CG_L | 0.28 (0.04) | 0.28 (0.04) | -1.759 | 0.778 |
| coupling_SFG_L | 0.19 (0.06) | 0.17 (0.06) | -0.688 | 0.027 |
| coupling_PrG_L | 0.14 (0.06) | 0.14 (0.05) | -1.890 | 0.798 |
| coupling_BG_R | 0.16 (0.08) | 0.16 (0.09) | 0.742 | 0.833 |
| coupling_SMN | 0.29 (0.04) | 0.29 (0.04) | -0.451 | 0.874 |

Abbreviations: SD, standard deviation; LASSO, Least Absolute Shrinkage and Selection Operator; BC, betweenness centrality; DC, degree centrality; NCP, nodal clustering coefficient; NLE, nodal local efficiency; NE, nodal efficiency; STG, superior temporal gyrus; SPL, superior parietal lobule; PCL, paracentral lobule; pSTS, posterior superior temporal sulcus; BG, basal ganglia; IPL, inferior parietal lobule; INS, insula; LOcC, lateral occipital cortex; IFG, inferior frontal gyrus; MFG, middle frontal gyrus; PoG, postcentral gyrus; CG, cingulate gyrus; SFG, superior frontal gyrus; PrG, precentral gyrus; SMN, somatomotor network.

### S4.2 Model Construction and Evaluation

Based on the 29 feature variables selected through LASSO regression, this study employed eight machine learning algorithms to construct predictive models, including SVM, GBM, Neural Network, XGBoost, KNN, AdaBoost, LightGBM, and CatBoost. Among all models, GBM demonstrated the best performance on the testing set, achieving an AUC value of 0.814 (95% confidence interval [CI]: 0.683-0.945). **Table S16** provides a detailed comparison of the performance of various machine learning models across multiple evaluation metrics for both training and testing sets. A comprehensive analysis of these metrics indicated that the GBM model exhibited the most superior overall performance. Results from the confusion matrix on the testing set showed that the GBM model achieved an accuracy of 76.2% and an F1-score of 73.7% (**Table S16**). The confusion matrices for the GBM model are presented in **Fig. S2**. The significance of the observed performance (*p_perm_* < 0.001) confirms that the model’s predictive power significantly exceeds chance levels and is not driven by over-fitting (**Fig. S3a**). Notably, the neuroimaging model substantially outperformed a clinical baseline model consisting of age and sex, which yielded a lower AUC of 0.644 (**Fig. S3b**). The feature importance ranking for GBM is illustrated in **Fig. S4.**

**Table S16. Performance comparison of machine learning models on training and testing sets**

| **Class** | **Model** | **Threshold** | **Accuracy** | **Sensitivity** | **Specificity** | **Precision** | **F1** |
| --- | --- | --- | --- | --- | --- | --- | --- |
| Training set | SVM | 0.377 | 0.738 | 0.81 | 0.667 | 0.708 | 0.756 |
|  | GBM | 0.529 | 0.762 | 0.667 | 0.857 | 0.824 | 0.737 |
|  | NeuralNetwork | 0.112 | 0.357 | 0.524 | 0.19 | 0.393 | 0.449 |
|  | Xgboost | 0.499 | 0.714 | 0.952 | 0.476 | 0.645 | 0.769 |
|  | KNN | 0.358 | 0.738 | 0.81 | 0.667 | 0.708 | 0.756 |
|  | Adaboost | 0.779 | 0.595 | 0.524 | 0.667 | 0.611 | 0.564 |
|  | LightGBM | 0.496 | 0.69 | 0.762 | 0.619 | 0.667 | 0.711 |
|  | CatBoost | 0.618 | 0.733 | 0.846 | 0.623 | 0.688 | 0.759 |
| Testing set | SVM | 0.377 | 0.738 | 0.81 | 0.667 | 0.708 | 0.756 |
|  | GBM | 0.529 | 0.762 | 0.667 | 0.857 | 0.824 | 0.737 |
|  | NeuralNetwork | 0.112 | 0.357 | 0.524 | 0.19 | 0.393 | 0.449 |
|  | Xgboost | 0.499 | 0.714 | 0.952 | 0.476 | 0.645 | 0.769 |
|  | KNN | 0.358 | 0.738 | 0.81 | 0.667 | 0.708 | 0.756 |
|  | Adaboost | 0.779 | 0.595 | 0.524 | 0.667 | 0.611 | 0.564 |
|  | LightGBM | 0.496 | 0.690 | 0.762 | 0.619 | 0.667 | 0.711 |
|  | CatBoost | 0.608 | 0.690 | 0.905 | 0.476 | 0.633 | 0.745 |

Abbreviations: SVM, Support Vector Machine; GBM, Gradient Boosting Machine; XGBoost, eXtreme Gradient Boosting; KNN, K-Nearest Neighbors; LightGBM, Light Gradient Boosting Machine; CatBoost, Categorical Boosting; AdaBoost, Adaptive Boosting.

**Fig. S2 Confusion matrices of the GBM model on (a) the training set and (b) the testing set.** Abbreviations: HCs, healthy controls; ALS-ES, early stage amyotrophic lateral sclerosis;

**Fig. S3 Validation and comparison of classification models.** (a) Permutation testing illustrates that the observed model performance is significantly higher than the null distribution, confirming predictive robustness. (b) Comparison of ROC curves shows that the neuroimaging-based model provides superior diagnostic performance relative to a clinical baseline model utilizing only demographic variables.

**Fig. S4 Feature Importance Ranking of GBM.** Abbreviations: L, left; R, right; BC, betweenness centrality; DC, degree centrality; NCP, nodal clustering coefficient; NLE, nodal local efficiency; NE, nodal efficiency; STG, superior temporal gyrus; SPL, superior parietal lobule; PCL, paracentral lobule; pSTS, posterior superior temporal sulcus; BG, basal ganglia; IPL, inferior parietal lobule; INS, insula; LOcC, lateral occipital cortex; IFG, inferior frontal gyrus; MFG, middle frontal gyrus; PoG, postcentral gyrus; CG, cingulate gyrus; SFG, superior frontal gyrus; PrG, precentral gyrus; SMN, somatomotor network.

### S4.3 Model Interpretability Analysis

To elucidate the predictive model, we applied SHAP to interpret the 29 features within the GBM model. The results identified the following five features as the most influential predictors: SC-FC coupling in the left precentral gyrus (coupling_PrG_L), degree centrality from fMRI in the left superior frontal gyrus (fMRI_DC_SFG_L), SC-FC coupling in the left superior frontal gyrus (coupling_SFG_L), intra-network coupling of the somatomotor network (coupling_SMN), and degree centrality from fMRI in the right inferior parietal lobule (fMRI_DC_IPL_R). **Fig. S5** provides an individualized explanation for a specific ALS-ES patient, showing that a value of 0.102 for coupling_PrG_L contributed positively to the prediction (SHAP value = +0.246). For practical application, we developed an interactive, web-based tool (https://ljx19954.shinyapps.io/early_als) that provides individualized risk assessment (**Fig. S6**). The risk stratification is defined as: High Risk (probability ≥ 70%), Medium Risk (probability 30%-70%), and Low Risk (probability < 30%).

**Fig. S5 Individualized feature analysis and interpretation based on SHAP values.** Abbreviations: L, left; R, right; BC, betweenness centrality; DC, degree centrality; NCP, nodal clustering coefficient; NLE, nodal local efficiency; NE, nodal efficiency; MFG, middle frontal gyrus; SFG, superior frontal gyrus; IFG, inferior frontal gyrus; IPL, inferior parietal lobule; LOcC, lateral occipital cortex; PoG, postcentral gyrus; PrG, precentral gyrus; BG, basal ganglia.

**Fig. S6 Web-based prediction model interface.** Abbreviations: fMRI, functional Magnetic Resonance Imaging; DC, degree centrality; IPL, inferior parietal lobule; R, right; NCP, nodal clustering coefficient; pSTS, posterior superior temporal sulcus; LOcC, lateral occipital cortex; L, left; NE, nodal efficiency; PoG, postcentral gyrus; CG, cingulate gyrus; SFG, superior frontal gyrus; ALS-ES, Early Stage Amyotrophic Lateral Sclerosis.

## S5 Neurochemical and Cognitive Correlates of SC-FC Coupling

Spatial correlation analysis identified significant associations between SC-FC coupling alterations and specific neurotransmitter systems. Significant negative correlations were observed with serotonin receptors 5-hydroxytryptamine 2A (5-HT2A) (*r* = -0.35, *p*_FDR_ = 0.007). At the cellular level, SC-FC alterations showed strong negative correlations with mitochondrial complex markers (complex II: *r* = -0.40; complex IV: *r* = -0.42; respiratory capacity: *r* = -0.45; all *p*_FDR_ = 0.004) (**Tables S17, S18**). Cognitive decoding analysis revealed that SC-FC decoupling spatially correlated with cortical systems supporting high-level cognition. The strongest negative associations were observed with sensory processing systems (*r* = -0.195), followed by medial prefrontal (mpfc: *r* = -0.189; dmpfc: *r* = -0.169) and motor-related regions (motor cortex: *r* = -0.161; sensorimotor cortex: r = -0.160), indicating colocalization of SC-FC disruption with networks underlying sensory, motor, and prefrontal cognitive functions (**Table S19**).

**Table S17. Correlations Between Neurotransmitter and the PLS1 Component**

| **Neurotransmitter** | ***r*** | ***p*_FDR_** |
| --- | --- | --- |
| 5HT1a | -0.153 | 0.193 |
| 5HT1b | -0.317 | 0.021 |
| 5HT2a | -0.353 | 0.007 |
| 5HT4 | 0.334 | 0.007 |
| 5HT6 | -0.182 | 0.091 |
| A4B2 | -0.044 | 0.692 |
| CB1 | -0.197 | 0.134 |
| CBF | -0.380 | 0.007 |
| CMRglu | -0.232 | 0.038 |
| COX1 | -0.278 | 0.079 |
| D1 | 0.103 | 0.338 |
| D2 | -0.154 | 0.136 |
| DAT | 0.192 | 0.087 |
| FDOPA | 0.083 | 0.475 |
| GABAa5 | -0.341 | 0.007 |
| GABAa | -0.183 | 0.095 |
| HDAC | -0.310 | 0.025 |
| KOR | -0.217 | 0.095 |
| M1 | -0.179 | 0.109 |
| MOR | 0.115 | 0.490 |
| MU | 0.106 | 0.577 |
| Microglia | -0.060 | 0.577 |
| NAT | -0.248 | 0.026 |
| NMDA | 0.019 | 0.861 |
| SERT | 0.029 | 0.779 |
| SV2A | -0.267 | 0.019 |
| VAChT | 0.152 | 0.155 |
| VMAT2 | 0.169 | 0.130 |
| mGluR5 | -0.312 | 0.019 |

Abbreviations: *p*_FDR_, false discovery rate corrected *p*-value; *r*, Pearson correlation coefficient; 5HT, serotonin receptor; A4B2, nicotinic acetylcholine receptor; CB1, cannabinoid receptor; CBF, cerebral blood flow; CMRglu, cerebral metabolic rate of glucose; COX, cytochrome c oxidase; D1/D2, dopamine receptors; DAT, dopamine transporter; FDOPA, fluorodopa uptake; GABAa, gamma-aminobutyric acid receptor; HDAC, histone deacetylase; KOR, kappa opioid receptor; M1, muscarinic acetylcholine receptor; MOR, mu opioid receptor; NAT, noradrenaline transporter; NMDA, N-methyl-D-aspartate receptor; SERT, serotonin transporter; SV2A, synaptic vesicle glycoprotein 2A; VAChT, vesicular acetylcholine transporter; VMAT2, vesicular monoamine transporter.

**Table S18. Cellular Correlations Between Cell-Type-Specific and Mitochondrial Marker Expression with the PLS1**

| **Cellular** | ***r*** | ***p*_FDR_** |
| --- | --- | --- |
| Glia-Astro | 0.122 | 0.589 |
| Glia-Endo | 0.061 | 0.673 |
| Glia-Micro | 0.259 | 0.128 |
| Glia-OPC | 0.178 | 0.438 |
| Glia-Oligo | -0.007 | 0.978 |
| mitochondria-Complex1-NADH | -0.326 | 0.008 |
| mitochondria-Complex2-SDH | -0.401 | 0.004 |
| mitochondria-Complex4-COx | -0.421 | 0.004 |
| mitochondria-RespiratoryCapacity | -0.447 | 0.004 |
| mitochondria-TissueDensity | -0.257 | 0.057 |
| mitochondria-TissueRespCapacity | -0.400 | 0.004 |
| neuron-Ex1-CortProject-L23 | 0.037 | 0.895 |
| neuron-Ex2-Granule-L34 | -0.428 | 0.004 |
| neuron-Ex3-Granule-L4 | -0.361 | 0.004 |
| neuron-Ex4- SubcortProject-L4 | -0.208 | 0.116 |
| neuron-Ex5-SubcortProject-L456 | -0.208 | 0.128 |
| neuron-Ex6-SubcortProject-L56 | -0.091 | 0.673 |
| neuron-Ex7-CorticothalProject | -0.149 | 0.505 |
| neuron-Ex8-CorticothalProject-L6 | -0.187 | 0.220 |
| neuron-ln1-VIP-RELN-NDNF-L12 | 0.005 | 0.978 |
| neuron-In2-VIP-L6 | 0.140 | 0.438 |
| neuron-ln3-VIP-RELN-L6 | -0.007 | 0.978 |
| neuron-ln4-RELN-NDNF-L13 | 0.028 | 0.895 |
| neuron-ln5-CCK-NOS-CALB-L23 | 0.039 | 0.895 |
| neuron-ln6-PVALB-L45 | -0.235 | 0.051 |
| neuron-ln7-SST-CALB-NPY-L 56 | 0.075 | 0.673 |
| neuron-ln8-SST-NOS-L6 | -0.156 | 0.331 |

Abbreviations: *p*_FDR_, false discovery rate corrected *p*-value; *r*, Pearson correlation coefficient; Astro, astrocyte; Endo, endothelial; Micro, microglia; OPC, oligodendrocyte precursor cell; Oligo, oligodendrocyte; NADH, nicotinamide adenine dinucleotide; SDH, succinate dehydrogenase; COx, cytochrome c oxidase; Ex, excitatory neuron; In/ln, inhibitory neuron; VIP, vasoactive intestinal polypeptide; RELN, reelin; NDNF, neuron-derived neurotrophic factor; CCK, cholecystokinin; NOS, nitric oxide synthase; CALB, calbindin; PVALB, parvalbumin; SST, somatostatin; NPY, neuropeptide Y.

**Table S19. Neurosymptom and Cognitive Term Correlations with PLS1**

| **Term** | **Pearson's r** | **Term** | **Pearson's r** |
| --- | --- | --- | --- |
| autobiographical memory | -0.031972 | mental states | -0.123632 |
| autobiographical | -0.038411 | referential | -0.123916 |
| tactile | -0.075186 | primary somatosensory | -0.125254 |
| theory mind | -0.076981 | somatosensory cortices | -0.125746 |
| touch | -0.085264 | movements | -0.125945 |
| muscle | -0.086933 | postcentral gyrus | -0.126243 |
| thinking | -0.090505 | finger | -0.133228 |
| mind tom | -0.090626 | somatosensory cortex | -0.136634 |
| m1 | -0.093666 | postcentral | -0.137193 |
| tom | -0.09446 | somatosensory | -0.139605 |
| posterior insula | -0.098675 | dorsomedial prefrontal | -0.140589 |
| network dmn | -0.102517 | beliefs | -0.141308 |
| moral | -0.103318 | primary motor | -0.141973 |
| personal | -0.103361 | si | -0.14421 |
| medial pfc | -0.108885 | movement | -0.149601 |
| mind | -0.109043 | dorsomedial | -0.151442 |
| self referential | -0.110525 | sensorimotor cortex | -0.159742 |
| dmn | -0.111571 | motor cortex | -0.160624 |
| mental state | -0.112064 | medial prefrontal | -0.163111 |
| primary sensorimotor | -0.115443 | cortex dmpfc | -0.166192 |
| primary secondary | -0.117522 | dmpfc | -0.169053 |
| secondary somatosensory | -0.119997 | dorsal medial | -0.169075 |
| sii | -0.121063 | cortex mpfc | -0.178522 |
| hand | -0.121805 | mpfc | -0.188597 |
| executed | -0.123293 | sensory | -0.195334 |

Abbreviations: PLS1, the first primary latent variable from the partial least squares analysis; *r*, Pearson correlation coefficient; DMN, default mode network; PFC, prefrontal cortex; DMPFC, dorsomedial prefrontal cortex; MPFC, medial prefrontal cortex; M1, primary motor cortex; S1, primary somatosensory cortex; SII, secondary somatosensory cortex.

## S6 Gene set enrichment analysis

**Fig. S7 GO enrichment analysis of PLS1- and PLS1+ genes.** (a) Circular visualization of the top GO terms enriched for PLS1- genes. The plot displays the top 6 terms, gene counts, P-values, and enrichment factors. (b) Bar plot illustrating the top enriched GO terms for PLS1- genes, categorized by Biological Process (BP), Cellular Component (CC), and Molecular Function (MF). (c) Circular visualization of the top GO terms enriched for PLS1+ genes. The plot displays the top 6 terms, gene counts, P-values, and enrichment factors. (d) Bar plot illustrating the top enriched GO terms for PLS1+ genes, categorized by BP, CC, and MF.

**Fig. S8 KEGG pathway enrichment analysis of PLS1- and PLS1+ genes.** (a) Significantly enriched KEGG pathways for PLS1- genes. (b) Significantly enriched KEGG pathways for PLS1+ genes.

## S7 Enrichment for specific cell types and cortical layers

Cortical layers enrichment analysis further demonstrated that PLS1- genes were significantly enriched in layers III (NES = -2.34, *p* < 0.05) and layers V (NES = -1.77, *p* = 0.03) (**Fig. S9a**). Conversely, the PLS1+ gene set showed no significant enrichment in any cortical layer (**Fig. S9b**). The cortical layer distributions for the PLS1- and PLS1+ groups are detailed in **Table S20**.

**Table S20. Functional Characterization and Cortical Layer Enrichment of PLS1**

| **Class** | **Description** | **setSize** | **enrichmentScore** | **NES** | ***p*** | ***p_FDR_*** | **qvalue** | **rank** |
| --- | --- | --- | --- | --- | --- | --- | --- | --- |
| PLS1- | L3 | 41 | -0.369 | -2.344 | 0.000 | 0.001 | 0.001 | 226 |
|  | L5 | 12 | -0.455 | -1.774 | 0.014 | 0.042 | 0.030 | 124 |
|  | L4 | 29 | -0.271 | -1.509 | 0.087 | 0.174 | 0.122 | 259 |
|  | L6 | 8 | 0.351 | 1.254 | 0.193 | 0.290 | 0.203 | 225 |
|  | L1 | 1 | -0.777 | -1.041 | 0.436 | 0.523 | 0.367 | 142 |
|  | L2 | 13 | -0.191 | -0.765 | 0.765 | 0.765 | 0.537 | 427 |
| PLS1+ | L2 | 7 | 0.274 | 0.847 | 0.661 | 0.887 | 0.887 | 125 |
|  | L6 | 74 | 0.096 | 0.733 | 0.793 | 0.887 | 0.887 | 71 |
|  | L1 | 18 | 0.136 | 0.634 | 0.887 | 0.887 | 0.887 | 126 |

Abbreviations: NES, normalized enrichment score; L1-L6, cortical layers 1 to 6; DMN, default mode network; DMPFC, dorsomedial prefrontal cortex; MPFC, medial prefrontal cortex; M1, primary motor cortex; S1, primary somatosensory cortex.

**Fig. S9 Cell-type and laminar enrichment of SC-FC decoupling genes.** (a-b) Bubble plot and GSEA profiles for PLS1- genes (negatively correlated with coupling), showing cell-type specific and laminar distribution (L1-L6). (c-d) Bubble plot and GSEA profiles for PLS1+ genes (positively correlated with coupling), highlighting their unique transcriptomic signatures across the cortical architecture.

## S8 Single-cell analysis

### S8.1 Gene Expression Analysis

To explore the cellular basis of the PLS1 components, we conducted a single-cell analysis using genes with the highest and lowest PLS1 z-scores in the GSE219281 dataset. FMN1 was identified as the gene with the most negative z-score within the PLS1- component. Cell-type enrichment analysis revealed that PLS1- genes were significantly enriched in ASC and MG. Correspondingly, in the GSE219281 dataset, FMN1 demonstrated significantly differential expression in both ASC and MG (**Fig. S10a-c**). This finding was validated in the GSE174332 dataset, where FMN1 also showed significantly decreased expression in MG cells (**Fig. S10g-i**). We also investigated SMIM30, the gene with the most positively z-score in the PLS1+ component. Cell-type enrichment analysis indicated that PLS1+ genes were enriched in GABAergic neurons. In dataset GSE219281, SMIM30 was significantly differentially expressed in inhibitory neurons (inh_neuron), with higher expression in ALS patients compared to HCs (**Fig. S10d-f**). However, in dataset GSE174332, SMIM30 expression in inhibitory neurons was significantly lower in ALS patients than in HCs (**Fig. S10j-l**). Due to this contradictory result, SMIM30 was excluded from further analysis.

**Fig. S10 Single-cell expression analysis of FMN1 and SMIM30 in ALS datasets.** (a-c) FMN1 expression in the GSE219281 dataset: (a) violin plots comparing FMN1 expression between ALS-ES patients and healthy controls (HCs) across different cell types, (b) ridge plots displaying the density distribution of FMN1-expressing cells, and (c) bar charts showing the proportion of FMN1-expressing cells in each cell population. (d-f) SMIM30 expression in the GSE219281 dataset: (d) violin plots comparing SMIM30 expression between ALS patients and HCs in neuronal subtypes, (e) ridge plots illustrating the density distribution of SMIM30-expressing cells, and (f) bar charts quantifying the proportion of SMIM30-expressing cells. (g-i) FMN1 expression in the GSE174332 dataset: (g) violin plots comparing FMN1 expression between ALS patients and HCs, (h) ridge plots showing FMN1 expression density across cell types, and (i) bar charts displaying the percentage of FMN1-positive cells. (j-l) SMIM30 expression in the GSE174332 dataset: (j) violin plots comparing SMIM30 expression in inhibitory neurons between ALS patients and HCs, (k) ridge plots depicting SMIM30 expression density, and (l) bar charts showing the proportion of SMIM30-expressing inhibitory neurons. **Abbreviations:** VLMC, vascular leptomeningeal cell; OPC, oligodendrocyte precursor cell; ODC, oligodendrocyte; MG, microglia; Inh_neuron, inhibitory neuron; Exc_neuron, excitatory neuron; ENDO, endothelial cell; ASC, astrocyte; C9-ALS, C9orf72-mediated amyotrophic lateral sclerosis; T_Cell, T lymphocyte; Mural, mural cell; In, inhibitory neuron; Fibro, fibroblast; Ex, excitatory neuron.

### S8.2 GO and KEGG Pathway Analysis

We then performed GO and KEGG pathway enrichment analysis for FMN1-associated differentially expressed genes in the MG cell population from dataset GSE219281. The results, detailed in **Table S21**, indicated significant enrichment in pathways related to myeloid leukocyte activation, immune response regulation, and phagocytosis (e.g., GO:0002274 "myeloid leukocyte activation", hsa04145 "Phagosome", hsa04666 "Fc gamma R-mediated phagocytosis").

**Table S21. GO and KEGG Pathway Enrichment Analysis for FMN1-associated Differentially Expressed Genes in Microglia from GSE219281**

| **category** | **ID** | **Description** | **Rich**  **Factor** | **Fold**  **Enrichment** | **Z-score** | ***p_FDR_*** |
| --- | --- | --- | --- | --- | --- | --- |
| BP | GO:0002274 | myeloid leukocyte activation | 0.110 | 9.575 | 12.585 | 2.87E-12 |
| BP | GO:0002768 | immune response-regulating cell surface | 0.101 | 8.768 | 10.643 | 3.12E-09 |
| BP | GO:0002443 | leukocyte mediated immunity | 0.131 | 11.441 | 11.252 | 5.88E-09 |
| BP | GO:0002697 | regulation of immune effector process | 0.098 | 8.530 | 9.776 | 4.29E-08 |
| BP | GO:0002696 | positive regulation of leukocyte activation | 0.139 | 12.132 | 10.705 | 5.76E-08 |
| CC | GO:0030139 | endocytic vesicle | 0.118 | 10.250 | 10.121 | 6.02E-08 |
| CC | GO:0009897 | external side of plasma membrane | 0.095 | 8.298 | 8.886 | 5.92E-07 |
| CC | GO:0030667 | secretory granule membrane | 0.110 | 9.575 | 8.856 | 2.16E-06 |
| CC | GO:0030666 | endocytic vesicle membrane | 0.100 | 8.713 | 8.354 | 4.76E-06 |
| CC | GO:0070821 | tertiary granule membrane | 0.092 | 7.993 | 7.913 | 9.67E-06 |
| MF | GO:0140375 | immune receptor activity | 0.102 | 8.911 | 8.033 | 1.34E-05 |
| MF | GO:0019955 | cytokine binding | 0.076 | 6.656 | 7.369 | 1.34E-05 |
| MF | GO:0035325 | Toll-like receptor binding | 0.116 | 10.102 | 8.176 | 1.87E-05 |
| MF | GO:0004896 | cytokine receptor activity | 0.062 | 5.417 | 6.680 | 3.16E-05 |
| MF | GO:0023023 | MHC protein complex binding | 0.061 | 5.334 | 6.607 | 3.46E-05 |
| KEGG | hsa05152 | Tuberculosis | 0.121 | 13.253 | 18.641 | 1.06E-21 |
| KEGG | hsa04145 | Phagosome | 0.092 | 10.054 | 16.891 | 2.74E-21 |
| KEGG | hsa04666 | Fc gamma R-mediated phagocytosis | 0.072 | 7.874 | 14.749 | 1.22E-18 |
| KEGG | hsa04380 | Osteoclast differentiation | 0.078 | 8.516 | 14.797 | 4.62E-18 |
| KEGG | hsa05140 | Leishmaniasis | 0.081 | 8.823 | 14.636 | 2.62E-17 |
| KEGG | hsa05150 | Staphylococcus aureus infection | 0.062 | 6.891 | 10.669 | 2.11E-10 |
| KEGG | hsa04611 | Platelet activation | 0.057 | 6.301 | 10.279 | 2.11E-10 |
| KEGG | hsa04662 | B cell receptor signaling pathway | 0.063 | 6.969 | 10.240 | 6.87E-10 |
| KEGG | hsa04640 | Hematopoietic cell lineage | 0.077 | 8.619 | 10.482 | 2.87E-09 |
| KEGG | hsa04659 | Th17 cell differentiation | 0.137 | 15.275 | 11.623 | 3.79E-08 |
| KEGG | hsa04610 | Complement and coagulation cascades | 0.097 | 10.429 | 11.408 | 6.29E-09 |
| KEGG | hsa05322 | Systemic lupus erythematosus | 0.091 | 9.734 | 10.179 | 1.77E-07 |
| KEGG | hsa04664 | Fc epsilon RI signaling pathway | 0.385 | 41.180 | 10.179 | 1.09E-05 |
| KEGG | hsa04062 | Chemokine signaling pathway | 0.093 | 9.934 | 8.566 | 3.13E-05 |
| KEGG | hsa04613 | Neutrophil extracellular trap formation | 0.162 | 17.362 | 9.673 | 8.98E-05 |

Abbreviations: GO, Gene Ontology; KEGG, Kyoto Encyclopedia of Genes and Genomes; BP, biological process; CC, cellular component; MF, molecular function; FDR, false discovery rate; MHC, major histocompatibility complex.

### S8.3 Cell Scoring Analysis

To further investigate the functional impact of FMN1, we performed a comprehensive cell scoring analysis on four FMN1-associated gene sets: "Leukocyte Activation", "Immune Receptor Signaling", "Membrane Vesicle Dynamics", and "FMN1 GTPase Signaling". The analysis revealed that the activity of these pathways was significantly altered, as evidenced by multiple complementary methods. AUCell analysis, which quantifies gene set activity at the single-cell level, demonstrated distinct spatial patterns of enrichment for each gene set across the UMAP landscape (**Fig. S11a-d**). This visual representation was corroborated by a quantitative comparison between ALS and HCs groups. The AUCell violin plots clearly showed significant differences in the score distributions for all four pathways (**Fig. S12a-d**), statistically confirming their dysregulation in the ALS condition (*p* < 0.05). Collectively, these results from module scoring, GSVA, and AUCell analyses consistently affirm that FMN1-associated biological processes, particularly those involved in immune activation and cellular dynamics, are significantly perturbed, highlighting their potential role in the pathophysiology of ALS.

**Fig. S11 AUCell UMAP Plots for FMN1-Associated Gene Sets.** (a) Leukocyte Activation gene set. (b) Immune Receptor Signaling gene set. (c) Membrane Vesicle Dynamics gene set.(d) FMN1 GTPase Signaling gene set. UMAP plots displaying the AUCell scores, which represent the activity of specific gene sets.

**Fig. S12 AUCell Violin Plots for FMN1-Associated Gene Sets.** (a) Leukocyte Activation gene set. (b) Immune Receptor Signaling gene set. (c) Membrane Vesicle Dynamics gene set. (d) FMN1 GTPase Signaling gene set. Violin plots comparing the distribution of AUCell scores for each gene set between ALS and HCs groups.
